# Factors Influencing Sport Persistence Along the Socio-Ecological Model—A Presentation of Sport Persistence Models Based on the Findings of a Representative Hungarian Sample

**DOI:** 10.3390/sports13040097

**Published:** 2025-03-25

**Authors:** Karolina Eszter Kovács

**Affiliations:** Faculty of Arts Institute of Psychology, University of Debrecen, 4032 Debrecen, Hungary; karolina92.kovacs@gmail.com

**Keywords:** sport persistence, socio-ecological model, intrapersonal factors, micro-system, meso-system, exo-system, macro-system

## Abstract

**Background/Objectives**: Sport persistence, defined as an athlete’s behavioural commitment to sport, is influenced by various psychological, social, and environmental factors. This study examines sport persistence using an ecological framework, incorporating Bronfenbrenner’s socio-ecological model and Bauman’s multi-level physical activity model. It aims to identify the key determinants of sport persistence among Hungarian athletes and differentiate the influencing factors for competitive and recreational athletes. **Methods**: The study analysed data from 1105 Hungarian (518 male, 557 female) secondary (*n* = 478) and university (*n* = 626) students who pursued competitive (*n* = 533) or recreational (*n* = 572) sports. A structured questionnaire assessed socio-demographic factors, sport-specific variables, psychological characteristics, and environmental influences. Statistical analyses were applied to identify predictors of sport persistence. **Results**: For competitive athletes, persistence was most strongly predicted by goal orientation (β = 0.322; *p* < 0.001), win orientation (β = 0.156; *p* = 0.001), resilience (β = 0.161; *p* < 0.001), and training frequency (β = 0.122; *p* = 0.017). In contrast, recreational athletes’ persistence was linked to task orientation (β = 0.092; *p* = 0.013), well-being (β = 0.092; *p* = 0.008), and social support (β = 0.084; *p* = 0.006). The father’s employment status had a positive effect on persistence, while broader socio-demographic factors were more relevant for recreational athletes. The role of micro-, exo-, and macro-system factors, such as peer or teacher support and sport infrastructure, was found to be limited in influencing persistence levels. **Conclusions**: Sport persistence is primarily driven by individual psychological factors rather than broader ecological influences. Competitive athletes show persistence through performance-oriented traits, while recreational athletes rely more on well-being and motivation. These findings suggest that tailored interventions could enhance sport persistence and performance.

## 1. Introduction

The notion of sport persistence, which denotes a commitment to athletic pursuits, merges performance with engagement, leading to an athlete’s devotion to their selected sport. This dedication involves the development of qualities such as adaptive coping strategies, positive personality characteristics, and resilience [1]. Thus, the athlete demonstrates not only persistent behaviour in their sport but also a deep qualitative commitment to it [2]. This encompasses addressing, processing, and leveraging the stressors linked to performance plateaus, setbacks, injuries, achievements, and favourable experiences [3,4,5]. The behaviour and performance associated with sport persistence are encapsulated within this concept [6,7]. It remains a relatively unexplored domain in global practice, as existing research generally concentrates on athletic habits, sports motivation, and engagement levels. Ultimately, sport persistence serves as a performance metric that reflects an individual’s sustained physical activity [8]. It refers to an athlete’s sustained commitment to engaging in sports over time, despite challenges, setbacks, or competing demands. It encompasses both behavioural persistence (continued participation in training and competition) and psychological commitment (motivation, resilience, and long-term goal orientation) [7].

During their school years, talented young athletes may drop out of sports due to a variety of individual psychological, social, and contextual influences before they achieve peak performance [9,10]. Factors such as gender, socio-economic status, and support from parents, coaches, and peers can predict both persistence in sports and the likelihood of dropping out during childhood [11,12]. Consequently, both sport persistence and dropout are recognised as complex and multifactorial phenomena [13], significantly shaped by diverse socio-cultural backgrounds, behavioural elements, personal traits, attitudes, types of sport, and motivations [13,14].

Persistence is worth investigating following the ecological models. The core premise of Bronfenbrenner’s socio-ecological model is that individuals are deeply intertwined with and shaped by their surroundings [15]. Specifically, Bronfenbrenner maintains that individual behaviour can be analysed through four environmental systems: micro-, meso, exo-, and macro-systems. These systems exist as nested layers, with the innermost layer signifying the self. The micro-system includes a network of close connections, such as family members, peers, neighbours, school classmates, and the workplace environment. The second layer is represented by the meso-system, which encompasses the interrelations among the components of the microsystem mentioned earlier. Hence, the meso-system pertains to the connections between various micro-systems. The exo-system serves as the third layer, referring to supportive environments where individuals play a passive role. Factors at the exo-system level that affect participation in sports encompass formal environments and the physical attributes of the sporting landscape, including community centres, parks, recreation centres, sports clubs, and facilities. The macro-system, which is the fourth and outermost layer of Bronfenbrenner’s model, signifies a broad consistency among the previously identified systems (micro-, meso-, and exo-systems) within the societal context as a whole.

Bronfenbrenner’s theory is one of the most influential and widely applied theories in the fields of human development and educational psychology [16]. However, some attempts have been made to provide a socio-ecological framework for sporting activity too. The model developed by Bauman et al. [17] employs a comprehensive multi-level framework that categorises all variables influencing physical activity into distinct levels. Its ecological nature allows for considering individual interactions and their social and physical contexts. The core idea is that a thorough understanding of the factors affecting physical activity enables the creation of multi-level action plans that are much more likely to be effective [18]. This model takes into account individual variables, including biological and psychological aspects, and facilitates an extensive exploration of micro-, meso-, exo-, and macro-variables [19,20,21]. While the interplay of political, environmental, and global factors is believed to affect sports participation significantly, these elements have only been examined in a limited manner thus far.

In general, socio-demographic and career-related background variables are seldom emphasised as factors contributing to sport persistence, despite the established understanding that a mix of parental support, cultural, and socio-economic elements most accurately forecasts childhood involvement in sports, following the frameworks of Bronfenbrenner and Bauman. Numerous studies have revealed the substantial impact of gender on sports persistence, frequently showing that male athletes demonstrate higher levels of persistence, which is consistent with previous research on long-term engagement in sports [12,22]. Furthermore, it is essential to evaluate the influence of career-related variables, especially regarding participation and persistence in elite sports, as sports biography and experience are of great significance for elite athletes. In our systematic literature review [23] centred on existing research regarding sport persistence, we identified nine categories of individual psychological factors: personality, motivation, orientation, learning and development, commitment, positive feelings, negative feelings, future benefits, and health. These categories and their associated variables represent the most commonly examined elements in sports participation, dropout, and persistence studies. Persistence, viewed as a beneficial variable, yields more significant advantages as its level rises, as long as it stays within an optimal range. As a result, it shows a positive correlation with other advantageous factors, such as coping flexibility, optimism, self-assessment, and self-image, while exhibiting a negative correlation with detrimental factors like anxiety, stress, pessimism, and narcissism. This relationship is valid regarding correlation and the influence direction; positive variables provide a supportive impact, while negative variables hinder persistence in sports [24,25]. Alexe et al. [26,27] also stated that intrinsic motivation, integrated regulation, and identified regulation positively predicted resilience, while introjected regulation, external regulation, and amotivation positively predicted burnout, which decreases the likelihood of sport persistence.

In relation to the micro-system, as per Bronfenbrenner’s classification, it is essential to highlight the importance of family, peers, and coaches, each contributing positively to sustained sporting behaviour [10,28,29]. Minghetti et al. [30] found that an intergenerational exercise intervention led to significant improvements in physical fitness and psychosocial well-being in children. These findings suggest that intergenerational exercise programs can be an effective strategy for promoting health and well-being in children, leading to increased sport persistence. The measurement of factors at the meso-system level, such as the family–school and family–coach relationships, is notably lacking in this field, although some attention has been given. For example, prior research has indicated that the connection between school and sports can directly affect persistence in sports [31]. Finally, with respect to macro-system factors, cultural expectations, subjective norms, and beliefs may significantly influence persistence in sports [32].

This research aims to explore the factors determining sport persistence in the ecological model. To this end, the following hypotheses were formulated.

**H1:**  *We hypothesise that the role of sport-specific variables will be more pronounced for competitive athletes, while the role of socio-demographic background will be less pronounced.*

**H2:**  *With respect to individual psychological characteristics, we hypothesise that the role of competition-specific variables (sport orientation, success perception, grit, resilience, and rumination) will be more dominant among competitive athletes.*

**H3:**  *It is assumed that the role of micro-, exo-, and macro-system-level variables is more pronounced among competitive athletes.*

The socio-ecological model provides a well-established framework for understanding how individual, interpersonal, and environmental factors interact to influence behaviour. Each level of the model (micro-, meso-, exo-, and macro-systems) was considered to ensure a holistic approach. Previous research highlights that sport persistence is shaped by a combination of personal psychological traits, social relationships, and structural opportunities [23], which provided the basis for the selection of the socio-demographic and psychological variables measured during the research.

## 2. Materials and Methods

### 2.1. Sample

In the quantitative research, we planned to collect data at the national level on young people aged between 14 and 25 who regularly participate in sport. In order to study the secondary school age group, the main objective was to include students in grades 9–13 in sports schools, as public education-type sports schools provide a suitable setting for studying both competitive and recreational athletes. On the one hand, the dual objectives of the sports school programme combine the promotion of sport and academic careers, providing appropriate opportunities for adolescents playing sports at a higher level. On the other hand, previous research has shown that a significant proportion of students attending sports schools are not competitive but recreational athletes, and that sports schools typically have several type classes (i.e., other profiles), making public education-type sports schools an appropriate setting for the development of recreational athletes as a control group. With regard to higher education institutions, the sports school line cannot be continued, as currently only the Hungarian University of Sports Science in Hungary can be considered as a public education-type sports school. However, the inclusion of a single higher education institution would not have allowed a comprehensive study. Therefore, the involvement of competitive sports students in higher education institutions was planned to be implemented through university sports clubs. When designing the research, the aim was to create a sample that was representative of gender, type of municipality, and type of school. However, it was not possible to limit the data collection to public education-type sports schools only, as there were several refusals from several institutions to allow data collection. Accordingly, the originally planned two-stage stratified sampling was not feasible, due to which snowball sampling was used in addition to direct contacts with institutions. Overall, the combination of institutional outreach and snowball sampling was used to maximise participation, while acknowledging the limitation that convenience sampling may still introduce some selection bias.

Data collection took place between 8 January 2024 and 20 June 2024. Our inclusion criteria were as follows: (1) engagement in regular (at least monthly) sports activities (regardless of the type—i.e., individual or team—and level of sporting activity—i.e., recreational or competitive); (2) being engaged in secondary or higher education studies; and (3) age between 14 and 25 years. Athletes below 14 years and above 25 years as well as those whose did not have any ongoing studies in secondary or tertiary education were excluded. A total of 1209 completions were received; after data cleaning (excluding respondents not meeting the inclusion criteria), data from 1105 respondents remained for analysis. The basic concept of the sample design was organised around the secondary school and university student population, and therefore, our sample presentation follows primarily this distribution. Overall, 43.3% of participants were in secondary education, and 56.7% were in tertiary education. The sample consisted of 478 secondary school students (43.3%) and 626 university students (56.7%), with a nearly balanced gender distribution (52.3% female, 46.9% male). Participants resided in various urban and rural settings, with the largest proportion living in county towns (37.3%), followed by small towns, villages, and the capital city. Parents’ education varied, with most mothers and fathers having either secondary or tertiary education (Table 1).

Regarding sport-related variables (Table 2), nearly 50% of the respondents engaged in sports multiple times a week, with higher participation rates observed among secondary school students. Individual sports (57.5%) were more common than team sports, and club membership was evenly split (49.5% club members, 50.5% non-members). Competitive athletes constituted 48.2% of the sample, with most competing at national or county levels, while the remaining 51.8% participated recreationally.

### 2.2. Instruments

The research was conducted using questionnaires that have demonstrated score validity and those under Hungarian validation (the adaptation of the questionnaire is on-going). Accordingly, the questionnaire consisted of the following units: a socio-demographic questionnaire, a block of sport- and health-specific questions, a block of questions related to academic performance, psychological measures (perception of success, sport orientation, sport persistence, sports anxiety, grit, resilience, well-being, vision, peer support, perfectionist climate, and human values), and questions on relational embeddedness. The survey could be completed online, via Google Forms. The average time spent on the survey was 40 min. All the instruments were applied at the same time.

#### 2.2.1. Socio-Demographic Questionnaire

At the beginning of our questionnaire, various socio-demographic questions were registered regarding gender, age, current level of education (secondary or higher) and grade, type of settlement, parents’ educational attainment, parents’ employment, and objective financial situation.

#### 2.2.2. Sport- and Health-Specific Issues

After the socio-demographic data, sport-specific data were queried: self-rated health (10-point Likert scale), self-assessed fitness status (10-point Likert scale), frequency of exercise (several times a day, daily, several times a week, once a week, monthly, or less often), sporting activity in an association (yes/no), weekly time spent in sports club training (hours), weekly time spent on individual training (hours), type of sport (individual or team), and sporting level (international competitions, national championships, county-level championships, local championships, or non-competitive).

#### 2.2.3. Questions on Academic Performance

Prior to the use of psychological measures, questions were included to map objective and subjective learning characteristics: grade point average of the previous school year, language exam (yes, no—if yes, how many), and subjective academic achievement.

#### 2.2.4. Perception of Success Questionnaire (PSQ, [33])

The Perception of Success Questionnaire is used to assess athletes’ success perceptions. The 12-item instrument has items rated on a 5-point Likert scale. The questionnaire contains two subscales (task- and ego-oriented perception of success). The instrument’s original reliability is high (Cronbach’s alpha: 0.89 and 0.91).

#### 2.2.5. Short Version Sport Orientation Questionnaire [34]

The questionnaire consists of 13 items and includes three subscales: win orientation, goal orientation, and competition. Win orientation measures willingness to win, and goal orientation focuses on sports goals through winning the game. Competition, on the other hand, measures competitive orientation through anticipation of the game, enjoyment of the game, and achievement of the goal of the game. The statements are rated by the respondents on a 5-point Likert scale. The validity of the measure was adequate for all subscales (Cronbach’s α = 0.86, 0.85, and 0.89).

#### 2.2.6. Sport Persistence Questionnaire [2]

The Sport Persistence Questionnaire is a 13-item questionnaire that measures sport persistence through a factor. Items are rated on a 5-point Likert scale. The reliability measured in the original survey is Cronbach’s α = 0.943. The minimum score on the overall index is 14 points, and the maximum score is 65 points. A higher score indicates higher persistence.

#### 2.2.7. Sport Anxiety Scale-2 (SAS-2, [35])

The Sports Anxiety Scale is a 15-item measure used to assess anxiety among athletes. The questionnaire includes three subscales: somatic anxiety, worry, and concentration disruption. Each dimension consists of 5 items. Respondents are asked to rate on a 4-point scale how they feel during or before sporting competitions. The original questionnaire is reliable (somatic anxiety: Cronbach’s α = 0.84; worry: Cronbach’s α = 0.89; concentration disturbance: Cronbach’s α = 0.84).

#### 2.2.8. Short Grit Scale [36] 

The instrument is designed to measure grit, a combination of passion and perseverance, through a single-factor question structure consisting of eight statements. The average of the scores obtained is used as the basis for assessment. The items are interpreted on a 5-point Likert scale. The questionnaire also contains four reverse items. The measure includes two subscales: consistency of interest (Cronbach’s α = 0.77) and perseverance of effort (Cronbach’s α = 0.82).

#### 2.2.9. Connor–Davidson Resilience Scale [37] 

To measure resilience, we used the 10-item Connor–Davidson Resilience Scale, a measure of successful coping with stress. Participants are asked to rate each statement on a 5-point Likert scale. The Cronbach’s alpha observed in the validation of the Hungarian version was 0.851. Based on the scores obtained in the questionnaire, a higher score indicates a higher degree of resilience.

#### 2.2.10. WHO Well-Being Inventory [38,39]

The questionnaire measures well-being through 5 statements. The instrument focuses on the general well-being of the respondents over the past two weeks. The statements are answered on a four-point Likert scale. The instrument does not contain a reverse item, with a higher score indicating a more favourable psychological state. The original reliability of the questionnaire is high, with Cronbach’s α = 0.85. Szegedi Tudományegyetem Bölcsészettudományi Kar Neveléstudományi Doktori Iskola.

#### 2.2.11. Self in the Future Scale [40]

The instrument consists of 15 items, which respondents are asked to rate on a 5-point Likert scale. The questionnaire consists of five subscales, which are as follows: positive future (Cronbach’s α = 0.89), control of the future (Cronbach’s α = 0.73), time management (Cronbach’s α = 0.86), lack of self-efficacy (Cronbach’s α = 0.81), and uncertainty about the future (Cronbach’s α = 0.73). A higher score indicates a more positive future image.

#### 2.2.12. Ruminative Response Style Questionnaire [41,42]

The tool measures the level of rumination in 10 items. Items are rated on a 4-point Likert scale. The questionnaire contains two subscales, the brooding and reflection subscales. The initial reliability of the questionnaire was adequate (brooding: Cronbach’s α = 0.71; reflection: Cronbach’s α = 0.73). Higher scores indicate a higher presence of ruminative thoughts.

#### 2.2.13. Short Scale of Youth’s Social Support (SSYSS, [43,44])

The questionnaire is an 18-item questionnaire that measures the level of support perceived from parents (5 items), peers (8 items), and teachers (5 items). The parent and teacher subscale scores between 5 and 25 points, and the peer subscale scores between 8 and 40 points. The questionnaire is a widely accepted, accurate, and valid measure of social support for young people in international practice. The reliability of the questionnaire was adequate (parental support: Cronbach’s α = 0.818; peer support: Cronbach’s α = 0.825; teacher support: Cronbach’s α = 0.835; total questionnaire: Cronbach’s α = 0.837).

#### 2.2.14. Perfectionistic Climate Questionnaire—Sport Version [45] 

This questionnaire is a measure of the perfectionist climate, asking respondents to rate each statement on a 5-point Likert scale. The 20 items of the questionnaire measure the climate across five subscales, which are expectations (McDonald’s omega = 0.88), criticism (McDonald’s omega = 0.74), control (McDonald’s omega = 0.74), conditional regard (McDonald’s omega = 0.88), and anxiousness (McDonald’s omega = 0.74). (In the case of the PCQ, the authors used the McDonald’s omega value instead of Cronbach’s α, so unlike the other measures, we cannot report the original Cronbach’s α values in this case.)

#### 2.2.15. Portrait Value Questionnaire [46]

This measure consists of 21 short human portraits, which respondents rate as important to them in terms of different values. Respondents indicate how similar the person described in that item is to them for each item. Respondents’ personal values can be inferred from the implicit values of the people they see as similar to them. The questionnaire contains 10 subscales: self-direction, achievement, hedonism, recognition, power, security, conformity, tradition, benevolence, and universalism. The statements are rated on a 6-point Likert scale. For the original measure, each scale’s reliability (Cronbach’s α) ranged from 0.15 to 0.85.

#### 2.2.16. Relational Embeddedness [47]

In this block of questions, as a non-validated psychological measure, respondents’ relationship systems were measured in two relationship domains. On the one hand, some questions asked whether the participant had a teacher (regardless of the level of education) with whom he/she could have conversations on different topics and problems. On the other hand, the specificity of interactions with immediate classmates/student mates was also measured by questions such as whether the respondent has a classmate/student mate with whom he/she can share specific study and leisure activities. The instrument is a tool of the CHERD-Hungary research group. The Centre for Higher Education Research and Development (CHERD) of the University of Debrecen conducts basic and applied research in the field of higher education, mainly in the border regions of Hungary, Romania, and Ukraine. In their interviews, they usually focus on the specificities of the relationship between students and teachers, which we applied ourselves to the present research as a senior researcher of the research team. The research team is led by Prof. Dr. Gabriella Pusztai, the research team’s website is https://cherd.unideb.hu (accessed on 9 February 2024).

### 2.3. Statistical Analysis

The data were collected in an Excel spreadsheet. Data analysis was performed using IBM SPSS 22.0 and Jamovi 2.3.28 statistical software. The distribution of the data was examined using normality tests, such as the Kolmogorov–Smirnov and Shapiro–Wilk tests. The data followed a non-normal distribution, so the use of non-parametric procedures was justified. Accordingly, the Mann–Whitney (two groups) and Kruskal–Wallis (three or more groups) tests were used for between-group comparisons. The *p*-value cutoff was *p* < 0.05. Measurement of the evolution of a dependent variable along two independent variables was analysed using Friedman’s test. Multicollinearity was examined using Variance Inflation Factor (VIF) values, with all variables remaining below the commonly accepted threshold of 10, indicating no severe multicollinearity issues. Homoscedasticity was checked through visual inspection of residual plots, and no significant heteroscedasticity was observed. Based on these diagnostics, multiple linear regression analyses were conducted to examine predictors of sport persistence. Explanatory variables were measured in six models: (1) socio-demographic variables, (2) sport-related variables, (3) individual psychological variables, (4) micro-system factors, (5) sports infrastructure, and (6) social/human values in the macro-system. To test which model has the best explanatory power, R and R² values were considered.

## 3. Results

### 3.1. Trends in Sport Persistence as a Function of Sporting Level

First, the combined role of sport participation level and socio-demographic variables in the development of sport persistence is discussed to provide the descriptive statistical background for deeper analysis (Table 3). Gender differences are not significant when taking into account the level of sport participation. Men’s persistence is higher in both groups compared to women. The difference between the two levels is more significant for women compared to men (χ^2^ = 1946; df = 2; *p* < 0.001). When the type of settlement and level of sport are taken into account together, an interesting trend in sport persistence is observed. Young people living in a small town context show higher sport persistence for competitive athletes compared to recreational athletes, while the opposite is true for young people living in metropolitan areas, where recreational athletes have a higher sport persistence compared to their competitive counterparts. The difference between groups is significant (χ^2^ = 1902; df = 2; *p* < 0.001).

Considering the level of sports participation and the parents’ educational level, the sports persistence advantage of children of mothers with secondary education is no longer clear. In the case of young people who participate in recreational sports, the highest sport persistence is indeed found among young people whose mother/carer has received secondary education; however, when looking at the competitive sports group, children of mothers with tertiary education had the highest sport persistence, compared to children of mothers with secondary education and children of mothers with primary education. A more homogeneous picture emerges with regard to the father’s educational attainment. The sport persistence scores of competitive athletes are higher than those of recreational athletes, but there is no dominance in either group based on the father’s educational attainment. In the recreational sports group, children of fathers with tertiary and primary education have almost identical scores, higher than the scores of those whose father/guardian received secondary education. In the competitive sub-sample, the children of fathers with tertiary and secondary education had almost identical persistence averages, while the persistence scores of young people whose father/guardian had primary education were lower. The difference between groups is significant for both the mother’s (χ^2^ = 2168; df = 2; *p* < 0.001) and father’s (χ^2^ = 2164; df = 2; *p* < 0.001) educational attainment.

Regarding the labour market status of parents, the trend in terms of sporting attainment is similar to the overall trend, with children of working parents having higher persistence rates in all groups, but the differences are not always striking. In the recreational sample, the average persistence score for children of working mothers is 45.7 points, which is higher compared to children of non-working mothers. The persistence score for children of working mothers is also higher in the competitive sample. The same findings apply to the labour market status of the father, both for the recreational and the competitive groups. Differences between groups are significant in both cases (mother’s labour market status: χ^2^ = 2013, df = 2, *p* < 0.001; father’s labour market status: χ^2^ = 2036, df = 2, *p* < 0.001).

When taking into account the type of sport and the level of sport in parallel, a different pattern from the general trend can be observed in the development of sport persistence values. If we look at the persistence values of individual and team athletes based on the level of sport, we can see that the competitive team athletes and individual sport athletes are almost identical, while the persistence of team sportsmen and women who play sports as a hobby is clearly higher than that of their individual sports counterparts. The difference between groups is significant (χ^2^ = 2065; df = 2; *p* < 0.001). The role of club sport membership in the development of sport persistence measures is also evident in terms of sport participation level, as both competitive and recreational athletes who participate in sports in club settings have significantly higher persistence compared to young people who participate in competitive and recreational sports in non-club settings (χ^2^ = 2134; df = 2; p < 0.001).

The distribution of sport persistence by sport type and by sport change shows a similar pattern to the sport type, as the persistence rates of young people changing sport (M = 54.5; SD = 8.6) and not changing sport (M = 54.3; SD = 8.6) are almost the same among competitive athletes, while there is a difference among recreational athletes. The difference between groups is significant (χ^2^ = 2065; df = 2; *p* < 0.001).

### 3.2. Investigation of Factors Affecting Sport Persistence in a Sub-Sample of Competitive Athletes

The linear regression analysis and Kruskal–Wallis tests illustrated earlier showed that the role of sport level can be significant. Therefore, examining the factors influencing sport persistence separately in sub-samples according to sport level is worthwhile, which was also separated in our hypotheses. Regression analyses were conducted in the same way for both the competitive and recreational subgroups. The analyses were first conducted for competitive athletes (expected β-values are presented in Table 4 and the related *p*-values in Table S1).

The first model analysed the role of socio-demographic background variables in the development of sport persistence. Only the father’s employment was significant, with the father’s regular work activity being a predictor of higher sport persistence. However, overall, the role of socio-demographic background was not significant. The first model’s R^2^ value of 0.043 suggests that socio-demographic variables account for 4.3% of the variance in sport persistence. The adjusted R^2^ is too small to be taken into account. However, the change in F-value of model 1 (from 0 to 2.363) is significant (*p* = 0.010).

The second model measured the effect of sport-specific variables on sport persistence. The results showed that the frequency of sports participation significantly positively predicted sport persistence, indicating that sports participation at least daily is more likely to lead to high sport persistence. Sporting activity at a club was also a significant positive predictor; higher sport persistence scores were more common among youth members of a sports club. Training as a sports club member was also a significant positive predictor of high sports persistence, suggesting that higher hours of sports activity within the club setting increased sports persistence. The type of sport and the amount of individual training did not indicate a significant effect. Compared to the previous model, including sport-specific variables removed the significant role of the father’s employment. The R^2^ of the second model is 0.113, i.e., sport-specific variables account for 11.3% of the variance in sport persistence. The adjusted R^2^ increased from 0.025 to 0.087, indicating a weak relationship. The change in the F-value of the second model (from 2.363 to 8.058) is significant (*p* < 0.001).

In the third model, individual psychological variables were included. The role of goal orientation and competition orientation in sport persistence was significant and positive, suggesting that higher goal and competition orientation is a predictor of high sport persistence. Resilience was also found to be a positive determinant of sport persistence. With respect to grit, consistent interest is a significant positive predictor of high sport persistence. Finally, regarding rumination, a significant adverse effect was found for a lower level of tearing compared to a higher level of sport persistence. For the other psychological variables included, no significant correlation was found. In the new model, there was no change in the role of socio-demographic background variables, but the effect of sport type appeared for sport-specific variables, predicting higher sport persistence among team athletes. In addition, the supportive role of training at the club was removed. The significant, influential role of sport frequency and intensity of sport club training remained. The R^2^ of the third model is 0.584, i.e., the model including individual variables explained 58.4% of the variance in sport persistence. The adjusted R^2^ increased from 0.087 to 0.555, indicating a strong model, but with a slight correction for unnecessary predictors. The change in the F-value of the third model (from 8.085 to 29.592) is significant (*p* < 0.001).

In the fourth model, the role of micro-system factors was analysed, but no significant positive effect was found for any of the variables. There was no change in the role of socio-demographic variables. Regarding sport-specific variables, a significant effect of sport frequency and sport type was still found. For the individual psychological variables, there was no shift compared to the effects detected in the previous model. The R^2^ of the fourth model is 0.595, i.e., the model explains 59.5% of the variance in sport persistence. The adjusted R^2^ increased from 0.555 to 0.557, indicating almost the same model fit, and a strong model, but with a slight correction for unnecessary predictors. The change in the F-value (from 29.592 to 1.126) is not significant (*p* = 0.336).

In the fifth model, sports infrastructure was included. However, the results showed that none of these variables significantly affected sports persistence. The role of socio-demographic variables remained insignificant. The previously shown effects for sport-specific variables remained. There were no changes for individual psychological as well as micro-systemic variables. The R^2^ of the fifth model is 0.598, i.e., this model also explains 59.8% of the variance in sport persistence. The adjusted R^2^ increased from 0.557 to 0.558, indicating a similarly strong model. The change in the F-value of the model (from 1.116 to 1.555) is not significant (*p* = 0.212).

Finally, the role of social/human values in the macro-system was analysed. However, no significant effect of any factor could be demonstrated for the variables analysed. With the inclusion of new variables, there was no change in the role of socio-demographic and sport-specific variables. The same can be said for the individual-specific and micro- and exo-system-specific variables. The R^2^ of the sixth model is 0.603, i.e., this model explains 60.3% of the variance in sport persistence. The adjusted R^2^ decreased from 0.558 to 0.554, indicating a weaker model. The change in the F-value of the model (from 1.555 to 0.622) is not significant (*p* = 0.795). The results suggest that this model best interprets the factors influencing sport persistence.

### 3.3. Investigation of Factors Influencing Sport Persistence in a Sub-Sample of Recreational Athletes

Following on from the competitive sub-sample, the recreational sub-sample was also examined for factors affecting sport persistence as outlined above (expected β-values are presented in Table 5 and the related *p*-values in Table S2). For the socio-demographic background variables examined in the first model, age, type of municipality of residence, and the presence of a sibling were found to be significant influencing factors. A negative directional prediction was found for age, with an increase in age corresponding to a decrease in sport persistence. At the same time, the presence of a sibling had an increasing effect on sports persistence. For the type of municipality, the role of larger municipalities was found to be supportive of sport persistence. The role of other socio-demographic background variables was not significant. The first model’s R^2^ value of 0.049 suggests that socio-demographic variables account for 4.9% of the variance in sport persistence. The adjusted R^2^ is too small to be taken into account. However, the change in F-value of the first model (from 0 to 2.865) is significant (*p* = 0.002). The second model examined the effect of sport-specific variables on sport persistence. The results show that sport frequency is a significant positive predictor of sport persistence, so at least daily sporting activity is a predictor of higher sport persistence in recreational athletes. The role of sport type is also significant, suggesting that team sport participants are more likely to have higher sport persistence. The role of sporting activity in a club was also a significant positive determinant, suggesting that higher sport persistence scores are more common among young people pursuing sports in a sports club. Individual training was also a significant positive predictor of high sport persistence, suggesting that higher hours of individual (non-club) sporting activity increase sport persistence. The amount of sport pursued in a sports club did not indicate a significant effect. Compared to the previous model, when including sport-specific variables, the significant role of type of municipality disappeared, but the negative effect of age and the positive effect of the presence of a sibling persisted. The R^2^ of the second model is 0.206, i.e., sport-specific variables account for 20.6% of the variance in sport persistence. The adjusted R^2^ increased from 0.032 to 0.184, indicating a weak relationship. The change in the F-value of the second model (from 2.865 to 21.987) is significant (*p* < 0.001).

In the third model, individual psychological variables were included. A significant positive correlation was found for self-orientation in terms of perceived success, suggesting that higher levels of task orientation contribute to higher sport persistence. With respect to sport orientation, the role of goal orientation was significant and positively skewed, suggesting that higher goal orientation is associated with higher sport persistence. Well-being was also found to be a positive determinant of sport persistence. With respect to grit, consistent interest and persistent effort are both significant positive predictors of high sport persistence. The other psychological variables included did not indicate a significant association. In the new model, age’s role in the socio-demographic background variables disappeared, while the effect of the presence of a sibling persisted. For sport-specific variables, the roles of sport type and club sport disappeared. The significant, influential role of sport frequency and individual training intensity remained. The R^2^ of the third model is 0.564, i.e., the model including individual variables explains 56.4% of the variance in sport persistence. The adjusted R^2^ increased from 0.184 to 0.536, indicating a strong model, but with a slight correction for unnecessary predictors. The change in the F-value of the third model (from 21.98 to 23.181) is significant (*p* < 0.001).

In the fourth model, the role of micro-system factors was examined, but as before, no significant positive effect was found for any of the variables. Including the new variables did not change the role of socio-demographic variables. For sport-specific variables, significant predictors remained for sport frequency and individual training intensity. For the individual psychological variables, there was no shift from the effects shown in the previous model. The R^2^ of the fourth model is 0.571, i.e., the model explains 57.1% of the variance in sport persistence. The adjusted R^2^ decreased from 0.536 to 0.533, indicating a slightly weaker model. The change in F-value (from 23.181 to 0.739) is not significant (*p* = 0.713).

In the fifth model, the role of sports infrastructure was examined, but the results show no significant effect of any of the variables on sport persistence. For the socio-demographic variables, only the role of siblings remained significant. For the sport-specific variables, sports participation frequency and individual training intensity were found as significant predictors. For the individual psychological variables, the previously significant effect of persistent effort was modified to insignificant, but the influential role of the other psychological variables remained. Finally, there was no change in the micro-system variables. The R^2^ of the fifth model is 0.572, i.e., this model also explains 57.2% of the variance in sport persistence. The adjusted R^2^ decreased from 0.533 to 0.532, indicating a slightly weaker model. The change in the F-value of the model (from 0.739 to 0.558) is not significant (*p* = 0.573).

Finally, the role of social/human values in the macro-system was reviewed. However, for the variables included, only the value of power showed a significant negative prediction, suggesting that the importance of power as a value may result in lower levels of sport persistence. The significant effect of the other factors could not be confirmed. The inclusion of new variables did not change the role of socio-demographic and sport-specific variables. In terms of individual psychological correlates, the role of well-being decreased, but the role of persistent effort became significant again for grit, with the effects of task orientation, goal orientation, and consistent interest remaining. In addition, there were no changes for the micro- and exo-system variables. The R^2^ of the sixth model is 0.585, i.e., this model also explains 58.5% of the variance in sport persistence. The adjusted R^2^ increased from 0.532 to 0.538, indicating a stronger model. The change in the F-value of the model (from 0.558 to 1.582) is not significant (*p* = 0.109). The results suggest that this model best explains the factors influencing sport persistence.

## 4. Discussion

The primary objective of the research was to explore the characteristics of sport persistence, the underlying factors, and the determinants of its formation, which were explicitly interpreted using an ecological model. To analyse the determinants of sport persistence, six models were used to examine the different determinants associated with each level of Bronfenbrenner’s general and Bauman’s sport-adapted ecological models ((1) socio-demographic, (2) sport-specific (3) individual psychological, (4) micro-system-related, (5) exo-system-related, and (6) macrosystem-related variables), using linear regression analysis. Regression analyses were also run separately on the competitive and recreational athlete samples.

Regarding socio-demographic variables, only the labour market status of the father was found to have a significant positive effect out of the socio-demographic variables for competitive athletes. In contrast, when analysed among recreational athletes, gender, age, type of municipality of residence, labour market status of the father, and the presence of a sibling were all found to be significant factors. The father’s employment positively affects sports persistence in both groups, as financial and psychological support is key to high sports performance and/or persistence [48]. However, the effect of other socio-demographic variables was less significant among competitive athletes, as these athletes tend to have higher levels of commitment and motivation, which counteract the effects of these variables [49,50]. However, socio-demographic factors play a greater role among recreational athletes, as these athletes are less committed, and other life priorities can significantly impact their sporting behaviour [6,7].

Regarding sport-specific variables, a significant positive effect was found for competitive athletes in the case of sport frequency and club sport activity, whereby sporting activity at least daily and in a sports club contributed to higher sport persistence. In contrast, for recreational athletes, a significant sport persistence-enhancing role was indicated for sport frequency, sport type, club sport activity, and individual training in the regression analysis, which showed that recreational athletes’ sport persistence was higher for sporting activity that takes place at least daily, team sports, club sports, and also high hours of individual training. For competitive athletes, the frequency of sporting activity and sports club membership provide the necessary framework and support to maintain a high level of sporting performance [51,52]. In contrast, for recreational athletes, daily sports participation, team sports, club membership, and individual training provide the necessary motivation and commitment to maintain sport persistence [53].

In terms of individual psychological variables, goal orientation, win orientation, and resilience played positive roles in predicting persistence among competitive athletes. In addition to these correlates, an adverse effect of rumination, with a related negative effect of explicit tearing, was found for ruminative performance, which has been shown to have a detrimental role in sport persistence levels, which may be due to the fact that repetitive negative thoughts can increase anxiety and stress and also reduce self-confidence and performance [54]. The roles of task-oriented perception of success, goal orientation, well-being, and grit in supporting persistence were significant for recreational athletes [55,56,57]. Thus, rumination is not significant for recreational athletes, nor is the role of competitive orientation, which is not surprising as these two psychological correlates are more likely to be variables associated with high levels of achievement, which are not necessarily inherent to recreational sport [58,59].

The results related to the micro- and exo-systems unanimously concluded that the role of support from parents, peers, teachers, and coaches as well as the role of sport infrastructure in developing sport persistence levels are insignificant. However, with regard to the social values examined in relation to the macro-system, a significant negative correlation was found for the value of power with sport persistence in the case of recreational sports, i.e., a higher level of power as a social value was coded as a predictor of lower sport persistence levels, which was not detected in the competitive sample and only appeared in the recreational sub-sample. Underlying the negative directional effect, we hypothesise that the value of power is not well matched to the social and recreational motivations of recreational sports [60]. For recreational athletes, sport is often based on social relationships and community support. The value of power, with its emphasis on self-assertion and influence over others, may conflict with community values and thus reduce engagement in sport [61].

**Figure 1:**
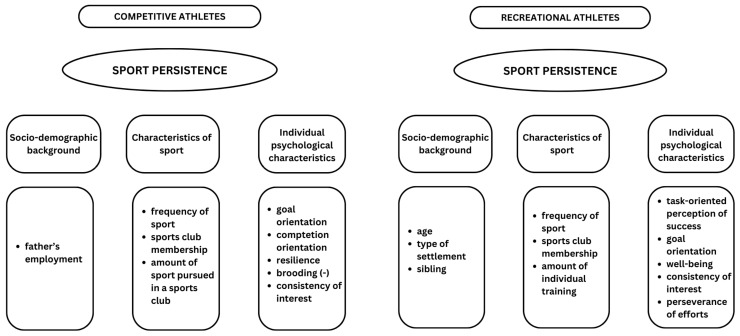
Factors determining the sport persistence of competitive and recreational athletes.

Overall, individual psychological effects best explain sport persistence, and therefore, socio-demographic, sport-specific, and individual psychological variables were considered in our models summarising the factors influencing sport persistence for the two sub-samples. The role of socio-demographic, sport-specific, and individual psychological variables was found to be dominant for sport persistence, but there were differences between competitive and recreational athletes (Figure 1). Socio-demographic background is more important for recreational athletes, especially age, type of settlement, and the presence of a sibling. In contrast, for competitive athletes, only the labour market status of the father is significant. Regarding the sport-specific variables, sports participation and club membership are both significant at the sport level. Nevertheless, in addition to these variables, the sport persistence of competitive athletes is explained by the amount of training in a sports club. In contrast, the sport persistence of recreational athletes is more predicted by the amount of training performed individually. As for individual psychological variables, the role of grit, including persistent effort, is consistent regardless of the level of sport participation, which is not surprising given that grit can be considered an analogous correlate to persistence. However, for competitive athletes, high goal and competition orientation and resilience, as well as low grit (as a ruminative component), are factors that determine sport persistence, which is not relevant for recreational athletes, where a sense of achievement and well-being play a more important role. This illustrates the different sporting focuses associated with sporting levels. Based on the results, hypothesis H1, which assumes that the role of sport-specific variables is more pronounced for competitive athletes while the role of socio-demographic background is less significant, is partially confirmed. The same can be stated for hypothesis H2, in which we hypothesised that the role of competition-specific variables would be more dominant among competitive athletes with respect to individual psychological characteristics. However, hypothesis H3, stating that the role of micro-, exo-, and macro-system-level variables is more predominant among competitive athletes, is not supported.

Among the limitations of the research, the sampling method should be highlighted. The initially planned two-stage stratified sampling was not feasible as most institutions refused to participate in the research. For this reason, snowball sampling was used, but this may distort the results and reduce their generalisability. A key limitation of this study is its cross-sectional design, which does not allow for causal inferences regarding the relationships between the examined variables. Future research employing longitudinal or experimental methodologies would be necessary to establish causal links in sport persistence. In addition, it should be mentioned that some questionnaires are not considered validated instruments in the country, and the primary objective of the research itself was not to validate the instruments. However, the study sample allows for the domestic validation of the non-validated questionnaires, which could be a further research direction. For some of the insignificant effects, it is suggested that using other instruments may be justified to measure certain influencing factors (e.g., peer support and social values). Furthermore, explanatory variables related to the meso- and macro-system levels are underrepresented in the research compared to individual and micro-system levels. However, the research findings suggest that individual psychological factors best explain sport persistence. System levels, which are distanced from the individual, are becoming less prominent in persistent sports behaviour, which is in line with our prior qualitative research [62,63].

## 5. Conclusions

The experience and results of the present research (together with our qualitative research) will allow us to create a sport persistence training programme that can effectively contribute to the performance and commitment of athletes, taking into account their individual strengths and weaknesses. Such training programmes can support student athletes’ perseverance in sport, with a focus on internal strengths, based on the research findings. This could provide sports professionals, coaches, and psychologists with a toolbox to enhance athletes’ performance and serve as a dropout prevention tool. Similar training programmes can be useful and effective when working with competitive athletes, for example, to reduce dropout tendencies and strengthen team relationships [14,64]. For recreational athletes, there is also the possibility of contextualising sporting support, which, in an academic context, could mean supporting secondary and tertiary studies through sport persistence [65]. In general, research can, therefore, contribute to a lasting commitment to sports among young people who participate in sports, which can have positive effects at both individual and societal levels, contributing to a healthy and productive lifestyle at the individual level and, in the long term, to the development of youth sporting habits that can project a picture of a healthier society. Strengthening the sport persistence of competitive athletes can lead to more effective participation in international sporting events, as well-designed sport psychology (sport persistence) training at the individual level can help individuals to overcome failed competitive experiences or sports injuries, leading to increased self-efficacy and performance, which can be reflected in national sports results over time.

## Figures and Tables

**Table 1:** Characteristics of the sample based on socio-demographic data (N = 1105).

		Secondary Level			Tertiary Level			*p*
		N	Row%	Adj Res	N	Row %	Adj Res	
Gender	Male	282	54.4%	7.0	236	45.6%	−7.0	<0.001
	Female	194	33.6%	−6.8	383	66.4%	6.8	
	I do not wish to answer	2	22.2%	−1.3	7	77.8%	1.3	
	Total	478	43.3%		626	56.7%		
Type of settlement	Capital	47	34.8%	−2.1	88	65.2%	2.1	<0.001
	County seat	153	37.1%	−3.2	259	62.9%	3.2	
	Big city	61	54.0%	2.4	52	46.0%	−2.4	
	Small town	145	55.3%	4.5	117	44.7%	−4.5	
	Village	67	39.0%	−1.3	105	61.0%	1.3	
	Farm	5	50.0%	0.4	5	50.0%	−0.4	
	Total	478	43.3%		626	56.7%		
Mother’s education	Primary	13	65.0%	2.0	7	35.0%	−2.0	<0.001
	Secondary	172	37.9%	−3.0	282	62.1%	3.0	
	Tertiary	275	44.9%	1.2	337	55.1%	−1.2	
	Does not know	18	100.0%	4.9	0	0.0%	−4.9	
	Total	478	43.3%		626	56.7%		
Father’s education	Primary	13	46.4%	0.3	15	53.6%	−0.3	<0.001
	Secondary	215	38.3%	−3.4	347	61.7%	3.4	
	Tertiary	231	46.7%	2.0	264	53.3%	−2.0	
	Does not know	19	100.0%	5.0	0	0.0%	−5.0	
	Total	478	43.3%		626	56.7%		

**Table 2:** Sample characteristics based on sport-related variables (N = 1105).

		Secondary			Tertiary			*p*
		N	Sor%	Adj Res	N	Sor%	Adj Res	
Exercise frequency	Several times a day	138	84.7%	11.5	25	15.3%	−11.5	<0.001
	Once a day	130	63.7%	6.5	74	36.3%	−6.5	
	Several times a week	181	33.1%	−6.8	366	66.9%	6.8	
	Once a week	16	15.5%	−6.0	87	84.5%	6.0	
	Monthly	13	14.9%	−5.6	74	85.1%	5.6	
	Total	478	43.3%		626	56.7%		
Type of sport	Team	305	65.0%	12.5	164	35.0%	−12.5	<0.001
	Individual	173	27.2%	−12.5	462	72.8%	12.5	
	Total	478	43.3%		626	56.7%		
Membership of an association	Yes	362	66.2%	15.2	185	33.8%	−15.2	<0.001
	No	116	20.8%	−15.2	441	79.2%	15.2	
	Total	478	43.3%		626	56.7%		
Sporting level/participation at competitions	Hobby	119	20.8%	−15.6	453	79.2%	15.6	<0.001
	Local, city competitions	18	32.1%	−1.7	38	67.9%	1.7	
	County championships	62	60.8%	3.7	40	39.2%	−3.7	
	International competitions	40	63.5%	3.3	23	36.5%	−3.3	
	National championships	239	76.8%	14.1	72	23.2%	−14.1	
	Total	478	43.3%		626	56.7%		

**Table 3:** The sport persistence of competitive and recreational athletes based on socio-demographic and sport-related variables.

	Competitive		Recreational	
Gender	M	SD	M	SD
Female	53.9	8.6	44.8	12.6
Male	54.8	12.5	46.5	12.3
Type of settlement				
Village or town	46.2	12.4	44.4	12.6
Bigger city or capital	55.1	8.8	54	8.8
Mother’s education				
Primary	46.3	16.3	40.3	15
Secondary	53.6	9	46.1	12.6
Tertiary	55.3	8.5	45.1	12.3
Father’s education				
Primary	50.2	14.5	46.8	10.9
Secondary	54.4	8.6	44.9	12.9
Tertiary	54.9	8.9	46.1	12.1
Mother’s employment				
Employed	54.6	8.6	45.7	12.6
Not employed	51.2	11.4	44.3	12.1
Father’s employment				
Employed	54.6	8.8	45.7	12.4
Not employed	47.4	12.9	43.2	12.1
Type of sport				
Individual	54.5	9.7	44.9	12.5
Team	54.3	8.5	48.5	10.7
Sports club membership				
Member	54.8	8.5	48.7	11.4
Not a member	50.3	10.8	45	12.6
Change in type of sport				
Yes	54.5	8.6	47.5	11.6
No	54.3	8.6	43.7	12.9

**Table 4:** Factors affecting sport persistence in a sample of competitive athletes (expected β-values, N = 1105).

	Model 1	Model 2	Model 3	Model 4	Model 5	Model 6
Gender	0.052	−0.008	−0.007	−0.011	−0.004	−0.009
Age	−0.132	−0.084	−0.088	−0.086	−0.084	−0.082
Level of study	0.066	0.096	0.064	0.066	0.063	0.063
Type of settlement	−0.042	−0.069	0.017	0.035	0.036	0.038
Mother’s education	0.039	0.074	0.081	0.085	0.084	0.086
Father’s education	−0.102	−0.152	−0.095	−0.101	−0.099	−0.101
Mother’s employment	0.082	0.082	0.056	0.058	0.057	0.058
Father’s employment	0.108 *	0.083	0.025	0.024	0.027	0.028
Change in family structure	−0.007	−0.028	−0.041	−0.036	−0.032	−0.032
Having a sibling	0.064	0.050	0.027	0.040	0.043	0.043
Exercise frequency		0.122 *	0.104 **	0.108 **	0.107 **	0.110 **
Type of sport		−0.038	−0.066 *	−0.066 *	−0.071 *	−0.072 *
Sports club membership		0.100 *	0.013	0.010	0.012	0.017
Training in a sports club (hours)		0.135 *	0.075 *	0.072	0.072	0.066
Individual training (hours)		0.087	0.032	0.037	0.034	0.032
PSQ task-orientation			0.029	0.036	0.044	0.041
PSQ ego-orientation			−0.036	−0.029	−0.037	−0.039
SOQ win-orientation			0.045	0.050	0.058	0.053
SOQ goal-orientation			0.322 ***	0.316 ***	0.317 ***	0.318 ***
SOQ competition			0.156 ***	0.166 ***	0.159 ***	0.166 ***
SAS-2 worry			0.010	0.009	0.005	0.003
SAS-2 somatic anxiety			−0.016	−0.020	−0.018	−0.019
SAS-2 concentration disruption			0.015	0.027	0.034	0.032
Well-being			0.036	0.029	0.031	0.025
SiF—positive future			0.017	0.021	0.023	0.023
SiF—control of the future			0.028	0.029	0.031	0.035
SiF—time management			0.006	−0.007	−0.002	−0.007
SiF—lack of self-efficacy			0.076	0.076	0.080	0.078
SiF—uncertainty about the future			−0.082	−0.080	−0.083	−0.085
Ruminations—brooding			−0.085 *	−0.083 *	−0.085 *	−0.095 *
Ruminations—reflections			0.065	0.055	0.054	0.056
Grit—consistency of interest			0.144 ***	0.147 ***	0.149 ***	0.142 **
Grit—perseverance of effort			0.045	0.044	0.042	0.041
SSYSS—peers				−0.073	−0.080	−0.082
SSYSS—teacher				0.087	0.088 *	0.082
SSYSS—parent				0.046	0.049	0.046
PCQ—expectations				−0.031	−0.035	−0.046
PCQ—criticism				−0.026	−0.017	−0.013
PCQ—control				0.024	0.022	0.009
PCQ—conditional regard				−0.015	−0.018	−0.014
PCQ—anxiousness				0.031	0.028	0.035
RE—personal support from teachers				0.022	0.020	0.009
RE—skill-focused support from teachers				0.066	0.061	0.073
RE—personal support from peers				−0.033	−0.026	−0.032
RE—skill-focused support from peers				−0.017	−0.019	−0.016
Participation in a school/university sports programme					0.057	0.053
Use of school/university sports infrastructure					−0.010	−0.003
PVQ self-direction						0.003
PVQ achievement						0.036
PVQ hedonism						−0.016
PVQ recognition						0.000
PVQ power						−0.010
PVQ security						−0.025
PVQ conformity						0.046
PVQ tradition						0.061
PVQ benevolence						0.010
PVQ universalism						−0.056
R	0.208	0.336	0.764	0.771	0.773	0.776
R^2^	0.043	0.113	0.584	0.595	0.598	0.603
Adjusted R^2^	0.025	0.087	0.555	0.557	0.558	0.554

* *p* < 0.05; ** *p* < 0.01; *** *p* < 0.001. Note: gender: 0 = female, 1 = male; education level: 0 = secondary, 1 = tertiary; type of settlement: 0 = maximum small town, 1 = minimum big city; mother and father’s education: 0 = maximum secondary, 1 = higher than secondary; mother and father’s employment: 0 = not employed, 1 = employed; family structure change: 0 = never had, 1 = had; sibling: 0 = none, 1 = at least one; sporting frequency: 0 = several times or less often per week, 1 = several times or more often per day; sporting level: 0 = recreational, 1 = competitive; sport type: 0 = individual, 1 = team; sports club membership: 0 = not a sports club member, 1 = sports club member; participation in school/university sports programme: 0 = used less than monthly at most, 1 = used more than monthly; school/university sports infrastructure: 0 = used monthly at most, 1 = used weekly at most. Age, training in a sports club (hours), individual training (hours), and individual psychological and micro- and macro-individual variables are continuous variables.

**Table 5:** Factors affecting sport persistence in a sample of recreational athletes (expected β-values, N = 1105).

	Model 1	Model 2	Model 3	Model 4	Model 5	Model 6
Gender	0.077	−0.009	0.016	0.025	0.023	0.033
Age	−0.178 **	−0.134 *	−0.076	−0.068	−0.062	−0.058
Level of study	0.068	0.093	−0.006	−0.018	−0.021	−0.025
Type of settlement	0.100 *	0.047	0.037	0.036	0.034	0.030
Mother’s education	−0.062	−0.067	−0.056	−0.065	−0.064	−0.066
Father’s education	0.028	0.030	0.040	0.040	0.041	0.050
Mother’s employment	0.009	−0.003	−0.019	−0.016	−0.014	−0.015
Father’s employment	0.034	−0.005	0.004	0.003	0.006	0.000
Change in family structure	−0.026	−0.040	−0.028	−0.022	−0.022	−0.027
Having a sibling	0.117 **	0.128 **	0.078 **	0.082 **	0.084 **	0.076 *
Exercise frequency		0.169 ***	0.084 *	0.081 *	0.081 *	0.080 *
Type of sport		0.090 *	0.034	0.034	0.037	0.032
Sports club membership		0.111 *	0.057	0.055	0.055	0.058
Training in a sports club (hours)		−0.039	0.002	−0.005	−0.005	−0.003
Individual training (hours)		0.317 ***	0.178 ***	0.186 ***	0.192 ***	0.195 ***
PSQ task-orientation			0.092 *	0.104 *	0.098 *	0.096 *
PSQ ego-orientation			0.055	0.051	0.045	0.047
SOQ win-orientation			0.005	0.000	0.003	0.026
SOQ goal-orientation			0.373 ***	0.364 ***	0.363 ***	0.350 ***
SOQ competition			−0.003	−0.001	0.005	−0.001
SAS-2 worry			−0.013	−0.011	−0.013	−0.013
SAS-2 somatic anxiety			−0.023	−0.027	−0.031	−0.038
SAS-2 concentration disruption			0.079	0.085	0.087	0.097
Well-being			0.092 **	0.082 *	0.083 *	0.064
SiF—positive future			−0.022	−0.028	−0.026	−0.031
SiF—control of the future			−0.004	−0.007	−0.007	−0.031
SiF—time management			0.027	0.028	0.029	0.033
SiF—lack of self-efficacy			0.006	−0.005	−0.006	−0.020
SiF—uncertainty about the future			−0.010	−0.006	−0.007	−0.015
Ruminations—brooding			0.005	0.002	0.007	0.013
Ruminations—reflections			0.060	0.061	0.060	0.058
Grit—consistency of interest			0.194 ***	0.186 ***	0.184 ***	0.175 ***
Grit—perseverance of effort			0.077 **	0.072 **	0.071	0.081 *
SSYSS—peers				0.002	0.004	0.007
SSYSS—teacher				0.021	0.024	0.017
SSYSS—parent				0.006	0.007	0.006
PCQ—expectations				0.083	0.083	0.075
PCQ—criticism				−0.045	−0.050	−0.053
PCQ—control				0.042	0.046	0.050
PCQ—conditional regard				−0.030	−0.028	−0.031
PCQ—anxiousness				−0.039	−0.036	−0.028
RE—personal support from teachers				−0.011	−0.006	−0.014
RE—skill-focused support from teachers				−0.053	−0.056	−0.057
RE—personal support from peers				0.037	0.037	0.034
RE—skill-focused support from peers				0.046	0.047	0.041
Participation in a school/university sports programme					−0.037	−0.036
Use of school/university sports infrastructure					0.021	0.024
PVQ self-direction						0.043
PVQ achievement						0.066
PVQ hedonism						0.029
PVQ recognition						−0.002
PVQ power						−0.087 *
PVQ security						0.018
PVQ conformity						0.042
PVQ tradition						−0.008
PVQ benevolence						−0.092
PVQ universalism						0.053
R	0.22	0.453	0.751	0.756	0.756	0.765
R^2^	0.049	0.206	0.564	0.571	0.572	0.585
Adjusted R^2^	0.032	0.184	0.536	0.533	0.532	0.538

* *p* < 0.05; ** *p* < 0.01; *** *p* < 0.001. Note: gender: 0 = female, 1 = male; education level: 0 = secondary, 1 = tertiary; type of settlement: 0 = maximum small town, 1 = minimum big city; mother and father’s education: 0 = maximum secondary, 1 = higher than secondary; mother and father’s employment: 0 = not employed, 1 = employed; family structure change: 0 = never had, 1 = had; sibling: 0 = none, 1 = at least one; sporting frequency: 0 = several times or less often per week, 1 = several times or more often per day; sporting level: 0 = recreational, 1 = competitive; sport type: 0 = individual, 1 = team; sports club membership: 0 = not a sports club member, 1 = sports club member; participation in school/university sports programme: 0 = used less than monthly at most, 1 = used more than monthly; school/university sports infrastructure: 0 = used monthly at most, 1 = used weekly at most. Age, training in a sports club (hours), individual training (hours), and individual psychological and micro- and macro-individual variables are continuous variables.

## Data Availability

Data are available only on request due to ethical restrictions.

## Supplementary Materials

The following supporting information can be downloaded at: https://www.mdpi.com/article/10.3390/sports13040097/s1.

## Abbreviations

The following abbreviations are used in this manuscript:

PSQ	Perception of Success Questionnaire
SOQ	Sport Orientation Questionnaire
SAS-2	Sport Anxiety Scale 2
SiF	Self in the Future
SSYSS	Short Scale of Youth’s Social Support
PSQ	Perfectionistic Climate Questionnaire
PVQ	Portrait Value Questionnaire
RE	Relational embeddedness
