# Supplementary material for: Factors Influencing Sport Persistence Along the Socio-Ecological Model—A Presentation of Sport Persistence Models Based on the Findings of a Representative Hungarian Sample
Source: Sports (Basel). 2025 Mar 25;13(4):97. doi: 10.3390/sports13040097 (PMC12030790; doi:10.3390/sports13040097)
Supplement: Supplementary file 1 [file sports-13-00097-s001.zip › sports-3501651-supplementary.pdf]

**Supplementary material**

Table A1. Results of linear regression analysis on the competitive athlete sample

| Model | | Unstandardized Coefficients | | Standardized Coefficients | | | Collinearity Statistics | |
|---|---|---|---|---|---|---|---|---|
| | | B | Std. Error | Beta | t | Sig. | Tolerance | VIF |
| 1 | (Constant) | 50.720 | 4.612 | | 10.997 | 0.000 | | |
| | Gender | 0.947 | 0.797 | 0.052 | 1.187 | 0.236 | 0.941 | 1.062 |
| | Age | -0.379 | 0.211 | -0.132 | -1.799 | 0.073 | 0.339 | 2.950 |
| | Level of study | 1.248 | 1.373 | 0.066 | 0.909 | 0.364 | 0.346 | 2.891 |
| | Type of settlement | -0.766 | 0.812 | -0.042 | -0.943 | 0.346 | 0.908 | 1.101 |
| | Mother's education | 0.020 | 0.054 | 0.039 | 0.360 | 0.719 | 0.159 | 6.279 |
| | Father's education | -0.050 | 0.053 | -0.102 | -0.954 | 0.341 | 0.159 | 6.288 |
| | Mother's employment | 2.916 | 1.558 | 0.082 | 1.871 | 0.062 | 0.959 | 1.042 |
| | Father's employment | 6.160 | 2.519 | 0.108 | 2.446 | 0.015 | 0.946 | 1.057 |
| | Change in family structure | -0.133 | 0.896 | -0.007 | -0.148 | 0.882 | 0.945 | 1.058 |
| | Having a sibling | 1.751 | 1.176 | 0.064 | 1.489 | 0.137 | 0.992 | 1.008 |
| 2 | (Constant) | 42.187 | 5.075 | | 8.312 | 0.000 | | |
| | Gender | -0.144 | 0.800 | -0.008 | -0.179 | 0.858 | 0.876 | 1.141 |
| | Age | -0.240 | 0.209 | -0.084 | -1.146 | 0.252 | 0.322 | 3.103 |
| | Level of study | 1.812 | 1.340 | 0.096 | 1.352 | 0.177 | 0.340 | 2.941 |
| | Type of settlement | -1.249 | 0.798 | -0.069 | -1.565 | 0.118 | 0.881 | 1.135 |
| | Mother's education | 0.038 | 0.053 | 0.074 | 0.710 | 0.478 | 0.157 | 6.372 |
| | Father's education | -0.075 | 0.052 | -0.152 | -1.452 | 0.147 | 0.157 | 6.378 |
| | Mother's employment | 2.931 | 1.511 | 0.082 | 1.940 | 0.053 | 0.955 | 1.047 |
| | Father's employment | 4.734 | 2.451 | 0.083 | 1.931 | 0.054 | 0.936 | 1.068 |
| | Change in family structure | -0.563 | 0.872 | -0.028 | -0.646 | 0.519 | 0.936 | 1.069 |
| | Having a sibling | 1.378 | 1.143 | 0.050 | 1.206 | 0.228 | 0.983 | 1.017 |
| | Exercise frequency | 0.840 | 0.352 | 0.122 | 2.388 | 0.017 | 0.664 | 1.507 |
| | Type of sport | -0.747 | 0.863 | -0.038 | -0.865 | 0.387 | 0.895 | 1.117 |
| | Sports club membership | 3.019 | 1.438 | 0.100 | 2.100 | 0.036 | 0.762 | 1.313 |
| | Training in a sports club (hours) | 0.276 | 0.107 | 0.135 | 2.570 | 0.010 | 0.624 | 1.602 |

| | | | | | | | | |
|---|---|---|---|---|---|---|---|---|
| | **Individual training (hours)** | 0.150 | 0.078 | 0.087 | 1.926 | 0.055 | 0.839 | 1.192 |
| **3** | **(Constant)** | 7.224 | 5.151 | | 1.402 | 0.161 | | |
| | **Gender** | -0.121 | 0.638 | -0.007 | -0.190 | 0.849 | 0.671 | 1.491 |
| | **Age** | -0.253 | 0.150 | -0.088 | -1.689 | 0.092 | 0.306 | 3.271 |
| | **Level of study** | 1.204 | 0.959 | 0.064 | 1.255 | 0.210 | 0.324 | 3.090 |
| | **Type of settlement** | 0.301 | 0.567 | 0.017 | 0.531 | 0.596 | 0.849 | 1.178 |
| | **Mother's education** | 0.041 | 0.037 | 0.081 | 1.102 | 0.271 | 0.155 | 6.456 |
| | **Father's education** | -0.047 | 0.036 | -0.095 | -1.289 | 0.198 | 0.154 | 6.492 |
| | **Mother's employment** | 1.995 | 1.066 | 0.056 | 1.872 | 0.062 | 0.936 | 1.069 |
| | **Father's employment** | 1.440 | 1.753 | 0.025 | 0.821 | 0.412 | 0.892 | 1.121 |
| | **Change in family structure** | -0.842 | 0.625 | -0.041 | -1.347 | 0.179 | 0.886 | 1.129 |
| | **Having a sibling** | 0.750 | 0.808 | 0.027 | 0.928 | 0.354 | 0.959 | 1.043 |
| | **Exercise frequency** | 0.720 | 0.253 | 0.104 | 2.848 | 0.005 | 0.625 | 1.600 |
| | **Type of sport** | -1.303 | 0.622 | -0.066 | -2.093 | 0.037 | 0.839 | 1.191 |
| | **Sports club membership** | 0.388 | 1.027 | 0.013 | 0.378 | 0.706 | 0.727 | 1.375 |
| | **Training in a sports club (hours)** | 0.153 | 0.077 | 0.075 | 1.983 | 0.048 | 0.587 | 1.704 |
| | **Individual training (hours)** | 0.055 | 0.056 | 0.032 | 0.977 | 0.329 | 0.792 | 1.263 |
| | **PSQ task-orientation** | 0.066 | 0.095 | 0.029 | 0.695 | 0.488 | 0.483 | 2.072 |
| | **PSQ ego-orientation** | -0.051 | 0.063 | -0.036 | -0.809 | 0.419 | 0.418 | 2.391 |
| | **SOQ win-orientation** | 0.077 | 0.087 | 0.045 | 0.891 | 0.373 | 0.330 | 3.027 |
| | **SOQ goal-orientation** | 1.034 | 0.163 | 0.322 | 6.348 | 0.000 | 0.325 | 3.076 |
| | **SOQ competition** | 0.597 | 0.173 | 0.156 | 3.451 | 0.001 | 0.409 | 2.443 |
| | **SAS-2 worry** | 0.019 | 0.086 | 0.010 | 0.226 | 0.822 | 0.399 | 2.504 |
| | **SAS-2-somatic anxiety** | -0.039 | 0.122 | -0.016 | -0.319 | 0.750 | 0.331 | 3.020 |
| | **SAS-2 concentration disruption** | 0.037 | 0.116 | 0.015 | 0.320 | 0.749 | 0.405 | 2.472 |
| | **Well-being** | 0.218 | 0.059 | 0.161 | 3.693 | 0.000 | 0.443 | 2.257 |
| | **SiF-positive future** | 0.075 | 0.078 | 0.036 | 0.965 | 0.335 | 0.614 | 1.628 |
| | **SiF – control of the future** | 0.037 | 0.090 | 0.017 | 0.414 | 0.679 | 0.480 | 2.081 |

| | | | | | | | | |
|---|---|---|---|---|---|---|---|---|
| | **SiF - time management** | 0.069 | 0.104 | 0.028 | 0.667 | 0.505 | 0.486 | 2.058 |
| | **SiF-lack of self-efficacy** | 0.012 | 0.071 | 0.006 | 0.164 | 0.870 | 0.602 | 1.660 |
| | **SiF - Uncertainty about the future** | 0.139 | 0.076 | 0.076 | 1.822 | 0.069 | 0.485 | 2.063 |
| | **Ruminations-brooding** | -0.206 | 0.124 | -0.082 | -1.661 | 0.097 | 0.341 | 2.931 |
| | **Ruminations-reflections** | -0.191 | 0.098 | -0.085 | -1.961 | 0.050 | 0.448 | 2.234 |
| | **Grit – consistency of interest** | 0.156 | 0.097 | 0.065 | 1.615 | 0.107 | 0.514 | 1.946 |
| | **Grit - perseverance of effort** | 0.389 | 0.103 | 0.144 | 3.782 | 0.000 | 0.576 | 1.735 |
| | **Gender** | 0.099 | 0.084 | 0.045 | 1.185 | 0.236 | 0.586 | 1.706 |
| **5** | **(Constant)** | 4.827 | 5.337 | | 0.904 | 0.366 | | |
| | **Gender** | -0.191 | 0.643 | -0.011 | -0.296 | 0.767 | 0.658 | 1.520 |
| | **Age** | -0.247 | 0.153 | -0.086 | -1.618 | 0.106 | 0.293 | 3.414 |
| | **Level of study** | 1.247 | 0.978 | 0.066 | 1.275 | 0.203 | 0.310 | 3.227 |
| | **Type of settlement** | 0.637 | 0.580 | 0.035 | 1.098 | 0.273 | 0.811 | 1.233 |
| | **Mother's education** | 0.043 | 0.037 | 0.085 | 1.155 | 0.249 | 0.153 | 6.529 |
| | **Father's education** | -0.050 | 0.037 | -0.101 | -1.351 | 0.177 | 0.151 | 6.627 |
| | **Mother's employment** | 2.060 | 1.073 | 0.058 | 1.921 | 0.055 | 0.921 | 1.086 |
| | **Father's employment** | 1.359 | 1.765 | 0.024 | 0.770 | 0.442 | 0.876 | 1.141 |
| | **Change in family structure** | -0.723 | 0.630 | -0.036 | -1.148 | 0.252 | 0.870 | 1.150 |
| | **Having a sibling** | 1.082 | 0.828 | 0.040 | 1.307 | 0.192 | 0.910 | 1.099 |
| | **Exercise frequency** | 0.747 | 0.256 | 0.108 | 2.920 | 0.004 | 0.608 | 1.644 |
| | **Type of sport** | -1.304 | 0.652 | -0.066 | -2.001 | 0.046 | 0.763 | 1.310 |
| | **Sports club membership** | 0.289 | 1.044 | 0.010 | 0.277 | 0.782 | 0.702 | 1.425 |
| | **Training in a sports club (hours)** | 0.147 | 0.078 | 0.072 | 1.877 | 0.061 | 0.569 | 1.758 |
| | **Individual training (hours)** | 0.063 | 0.057 | 0.037 | 1.108 | 0.268 | 0.768 | 1.302 |
| | **PSQ task-orientation** | 0.083 | 0.096 | 0.036 | 0.861 | 0.390 | 0.467 | 2.140 |
| | **PSQ ego-orientation** | -0.041 | 0.067 | -0.029 | -0.619 | 0.536 | 0.377 | 2.651 |
| | **SOQ win-orientation** | 0.087 | 0.089 | 0.050 | 0.977 | 0.329 | 0.314 | 3.183 |

| | | | | | | | | |
|---|---|---|---|---|---|---|---|---|
| | **SOQ goal-orientation** | 1.013 | 0.166 | 0.316 | 6.111 | 0.000 | 0.313 | 3.193 |
| | **SOQ competition** | 0.635 | 0.176 | 0.166 | 3.599 | 0.000 | 0.393 | 2.545 |
| | **SAS-2 worry** | 0.018 | 0.089 | 0.009 | 0.201 | 0.841 | 0.375 | 2.663 |
| | **SAS-2-somatic anxiety** | -0.048 | 0.124 | -0.020 | -0.386 | 0.700 | 0.320 | 3.125 |
| | **SAS-2 concentration disruption** | 0.069 | 0.120 | 0.027 | 0.574 | 0.566 | 0.379 | 2.641 |
| | **Well-being** | 0.212 | 0.060 | 0.157 | 3.544 | 0.000 | 0.427 | 2.340 |
| | **SiF-positive future** | 0.062 | 0.079 | 0.029 | 0.784 | 0.433 | 0.598 | 1.671 |
| | **SiF – control of the future** | 0.045 | 0.091 | 0.021 | 0.498 | 0.619 | 0.469 | 2.133 |
| | **SiF - time management** | 0.072 | 0.106 | 0.029 | 0.681 | 0.496 | 0.463 | 2.160 |
| | **SiF-lack of self-efficacy** | -0.012 | 0.074 | -0.007 | -0.168 | 0.867 | 0.556 | 1.798 |
| | **SiF - Uncertainty about the future** | 0.139 | 0.078 | 0.076 | 1.793 | 0.074 | 0.465 | 2.150 |
| | **Ruminations-brooding** | -0.201 | 0.125 | -0.080 | -1.605 | 0.109 | 0.333 | 3.000 |
| | **Ruminations-reflections** | -0.187 | 0.100 | -0.083 | -1.881 | 0.061 | 0.428 | 2.337 |
| | **Grit – consistency of interest** | 0.132 | 0.098 | 0.055 | 1.346 | 0.179 | 0.497 | 2.010 |
| | **Grit - perseverance of effort** | 0.396 | 0.108 | 0.147 | 3.669 | 0.000 | 0.523 | 1.911 |
| | **SSYSS - peers** | 0.098 | 0.087 | 0.044 | 1.122 | 0.263 | 0.538 | 1.858 |
| | **SSYSS - teacher** | -0.073 | 0.045 | -0.073 | -1.634 | 0.103 | 0.416 | 2.403 |
| | **SSYSS - parent** | 0.124 | 0.064 | 0.087 | 1.951 | 0.052 | 0.423 | 2.365 |
| | **PCQ - expectations** | 0.069 | 0.059 | 0.046 | 1.169 | 0.243 | 0.548 | 1.825 |
| | **PCQ - criticism** | -0.058 | 0.098 | -0.031 | -0.595 | 0.552 | 0.306 | 3.270 |
| | **PCQ - control** | -0.047 | 0.111 | -0.026 | -0.421 | 0.674 | 0.227 | 4.405 |
| | **PCQ – conditional regard** | 0.046 | 0.105 | 0.024 | 0.437 | 0.662 | 0.269 | 3.716 |
| | **PCQ - anxiousness** | -0.028 | 0.106 | -0.015 | -0.262 | 0.793 | 0.244 | 4.100 |
| | **RE – personal support from teachers** | 0.059 | 0.100 | 0.031 | 0.592 | 0.554 | 0.299 | 3.348 |
| | **RE – skill-focused support from teachers** | 0.080 | 0.169 | 0.022 | 0.473 | 0.636 | 0.369 | 2.707 |
| | **RE – personal support from peers** | 0.265 | 0.190 | 0.066 | 1.394 | 0.164 | 0.376 | 2.663 |

| | | | | | | | | |
|---|---|---|---|---|---|---|---|---|
| | **RE – skill-focused support from peers** | -0.091 | 0.147 | -0.033 | -0.616 | 0.538 | 0.289 | 3.455 |
| | **participation in a school/university sports programme** | -0.042 | 0.135 | -0.017 | -0.308 | 0.758 | 0.288 | 3.470 |
| | **use of school/university sports infrastructure** | 3.133 | 5.424 | | 0.578 | 0.564 | | |
| | **Gender** | -0.074 | 0.650 | -0.004 | -0.114 | 0.909 | 0.643 | 1.556 |
| | **Age** | -0.239 | 0.153 | -0.084 | -1.565 | 0.118 | 0.292 | 3.422 |
| | **Level of study** | 1.191 | 0.984 | 0.063 | 1.210 | 0.227 | 0.306 | 3.272 |
| | **Type of settlement** | 0.650 | 0.579 | 0.036 | 1.122 | 0.262 | 0.811 | 1.233 |
| | **Mother's education** | 0.043 | 0.037 | 0.084 | 1.144 | 0.253 | 0.153 | 6.530 |
| | **Father's education** | -0.049 | 0.037 | -0.099 | -1.338 | 0.182 | 0.151 | 6.627 |
| | **Mother's employment** | 2.046 | 1.071 | 0.057 | 1.909 | 0.057 | 0.920 | 1.086 |
| | **Father's employment** | 1.521 | 1.766 | 0.027 | 0.861 | 0.390 | 0.873 | 1.145 |
| | **Change in family structure** | -0.643 | 0.631 | -0.032 | -1.020 | 0.308 | 0.865 | 1.156 |
| | **Having a sibling** | 1.168 | 0.829 | 0.043 | 1.409 | 0.159 | 0.906 | 1.104 |
| | **Exercise frequency** | 0.739 | 0.256 | 0.107 | 2.888 | 0.004 | 0.608 | 1.645 |
| | **Type of sport** | -1.398 | 0.654 | -0.071 | -2.138 | 0.033 | 0.756 | 1.322 |
| | **Sports club membership** | 0.352 | 1.044 | 0.012 | 0.337 | 0.736 | 0.700 | 1.429 |
| | **Training in a sports club (hours)** | 0.148 | 0.078 | 0.072 | 1.886 | 0.060 | 0.568 | 1.760 |
| | **Individual training (hours)** | 0.058 | 0.057 | 0.034 | 1.019 | 0.309 | 0.763 | 1.311 |
| | **PSQ task-orientation** | 0.101 | 0.097 | 0.044 | 1.044 | 0.297 | 0.461 | 2.169 |
| | **PSQ ego-orientation** | -0.053 | 0.067 | -0.037 | -0.790 | 0.430 | 0.373 | 2.680 |
| | **SOQ win-orientation** | 0.099 | 0.089 | 0.058 | 1.117 | 0.265 | 0.312 | 3.205 |
| | **SOQ goal-orientation** | 1.016 | 0.166 | 0.317 | 6.138 | 0.000 | 0.313 | 3.195 |
| | **SOQ competition** | 0.609 | 0.177 | 0.159 | 3.444 | 0.001 | 0.390 | 2.564 |
| | **SAS-2 worry** | 0.010 | 0.089 | 0.005 | 0.111 | 0.912 | 0.374 | 2.671 |
| | **SAS-2-somatic anxiety** | -0.044 | 0.123 | -0.018 | -0.356 | 0.722 | 0.320 | 3.127 |

| | | | | | | | | |
|---|---|---|---|---|---|---|---|---|
| | **SAS-2 concentration disruption** | 0.086 | 0.120 | 0.034 | 0.713 | 0.476 | 0.376 | 2.659 |
| | **Well-being** | 0.214 | 0.060 | 0.158 | 3.566 | 0.000 | 0.427 | 2.342 |
| | **SiF-positive future** | 0.065 | 0.079 | 0.031 | 0.826 | 0.409 | 0.598 | 1.673 |
| | **SiF – control of the future** | 0.049 | 0.091 | 0.023 | 0.535 | 0.593 | 0.468 | 2.135 |
| | **SiF - time management** | 0.077 | 0.106 | 0.031 | 0.729 | 0.467 | 0.462 | 2.163 |
| | **SiF-lack of self-efficacy** | -0.005 | 0.074 | -0.002 | -0.062 | 0.950 | 0.548 | 1.825 |
| | **SiF - Uncertainty about the future** | 0.147 | 0.078 | 0.080 | 1.895 | 0.059 | 0.463 | 2.158 |
| | **Ruminations-brooding** | -0.208 | 0.125 | -0.083 | -1.668 | 0.096 | 0.333 | 3.006 |
| | **Ruminations-reflections** | -0.191 | 0.100 | -0.085 | -1.915 | 0.056 | 0.427 | 2.342 |
| | **Grit – consistency of interest** | 0.128 | 0.098 | 0.054 | 1.309 | 0.191 | 0.497 | 2.013 |
| | **Grit - perseverance of effort** | 0.401 | 0.108 | 0.149 | 3.705 | 0.000 | 0.518 | 1.930 |
| | **SSYSS - peers** | 0.094 | 0.087 | 0.042 | 1.070 | 0.285 | 0.535 | 1.868 |
| | **SSYSS - teacher** | -0.080 | 0.045 | -0.080 | -1.776 | 0.076 | 0.409 | 2.445 |
| | **SSYSS - parent** | 0.126 | 0.064 | 0.088 | 1.976 | 0.049 | 0.422 | 2.370 |
| | **PCQ - expectations** | 0.075 | 0.059 | 0.049 | 1.257 | 0.209 | 0.546 | 1.830 |
| | **PCQ - criticism** | -0.065 | 0.098 | -0.035 | -0.660 | 0.510 | 0.304 | 3.289 |
| | **PCQ - control** | -0.030 | 0.112 | -0.017 | -0.272 | 0.786 | 0.223 | 4.475 |
| | **PCQ – conditional regard** | 0.041 | 0.105 | 0.022 | 0.393 | 0.695 | 0.265 | 3.767 |
| | **PCQ - anxiousness** | -0.032 | 0.106 | -0.018 | -0.301 | 0.763 | 0.243 | 4.109 |
| | **RE – personal support from teachers** | 0.052 | 0.100 | 0.028 | 0.525 | 0.600 | 0.298 | 3.353 |
| | **RE – skill-focused support from teachers** | 0.070 | 0.169 | 0.020 | 0.418 | 0.676 | 0.368 | 2.716 |
| | **RE – personal support from peers** | 0.248 | 0.190 | 0.061 | 1.302 | 0.194 | 0.374 | 2.673 |
| | **RE – skill-focused support from peers** | -0.070 | 0.147 | -0.026 | -0.476 | 0.634 | 0.287 | 3.480 |
| | **participation in a school/university** | -0.047 | 0.136 | -0.019 | -0.349 | 0.727 | 0.287 | 3.480 |

| | | | | | | | | |
|---|---|---|---|---|---|---|---|---|
| | sports programme | | | | | | | |
| | use of school/university sports infrastructure | 0.377 | 0.220 | 0.057 | 1.711 | 0.088 | 0.741 | 1.350 |
| | Gender | -0.047 | 0.158 | -0.010 | -0.297 | 0.766 | 0.712 | 1.405 |
| 6 | (Constant) | 2.930 | 5.511 | | 0.532 | 0.595 | | |
| | Gender | -0.161 | 0.670 | -0.009 | -0.241 | 0.810 | 0.610 | 1.640 |
| | Age | -0.234 | 0.154 | -0.082 | -1.522 | 0.129 | 0.290 | 3.449 |
| | Level of study | 1.187 | 0.993 | 0.063 | 1.195 | 0.233 | 0.303 | 3.303 |
| | Type of settlement | 0.693 | 0.590 | 0.038 | 1.174 | 0.241 | 0.788 | 1.270 |
| | Mother's education | 0.043 | 0.038 | 0.086 | 1.155 | 0.249 | 0.153 | 6.541 |
| | Father's education | -0.050 | 0.037 | -0.101 | -1.358 | 0.175 | 0.151 | 6.633 |
| | Mother's employment | 2.049 | 1.091 | 0.058 | 1.879 | 0.061 | 0.896 | 1.117 |
| | Father's employment | 1.577 | 1.787 | 0.028 | 0.882 | 0.378 | 0.860 | 1.163 |
| | Change in family structure | -0.641 | 0.642 | -0.032 | -0.999 | 0.318 | 0.844 | 1.185 |
| | Having a sibling | 1.176 | 0.839 | 0.043 | 1.401 | 0.162 | 0.890 | 1.123 |
| | Exercise frequency | 0.760 | 0.260 | 0.110 | 2.927 | 0.004 | 0.594 | 1.684 |
| | Type of sport | -1.426 | 0.663 | -0.072 | -2.152 | 0.032 | 0.743 | 1.347 |
| | Sports club membership | 0.524 | 1.070 | 0.017 | 0.490 | 0.624 | 0.672 | 1.488 |
| | Training in a sports club (hours) | 0.134 | 0.080 | 0.066 | 1.681 | 0.093 | 0.552 | 1.811 |
| | Individual training (hours) | 0.056 | 0.058 | 0.032 | 0.962 | 0.337 | 0.742 | 1.348 |
| | PSQ task-orientation | 0.094 | 0.098 | 0.041 | 0.956 | 0.339 | 0.451 | 2.215 |
| | PSQ ego-orientation | -0.056 | 0.069 | -0.039 | -0.804 | 0.422 | 0.354 | 2.829 |
| | SOQ win-orientation | 0.091 | 0.091 | 0.053 | 1.003 | 0.316 | 0.301 | 3.319 |
| | SOQ goal-orientation | 1.021 | 0.169 | 0.318 | 6.060 | 0.000 | 0.305 | 3.284 |
| | SOQ competition | 0.635 | 0.180 | 0.166 | 3.520 | 0.000 | 0.377 | 2.650 |
| | SAS-2 worry | 0.006 | 0.091 | 0.003 | 0.067 | 0.947 | 0.358 | 2.797 |
| | SAS-2-somatic anxiety | -0.046 | 0.125 | -0.019 | -0.366 | 0.714 | 0.312 | 3.204 |
| | SAS-2 concentration disruption | 0.082 | 0.122 | 0.032 | 0.676 | 0.499 | 0.369 | 2.709 |
| | Well-being | 0.207 | 0.061 | 0.152 | 3.368 | 0.001 | 0.410 | 2.436 |

| | | | | | | | | |
|---|---|---|---|---|---|---|---|---|
| | **SiF-positive future** | 0.053 | 0.081 | 0.025 | 0.651 | 0.515 | 0.574 | 1.742 |
| | **SiF – control of the future** | 0.049 | 0.092 | 0.023 | 0.528 | 0.598 | 0.461 | 2.170 |
| | **SiF - time management** | 0.089 | 0.108 | 0.035 | 0.820 | 0.412 | 0.449 | 2.228 |
| | **SiF-lack of self-efficacy** | -0.014 | 0.077 | -0.007 | -0.184 | 0.854 | 0.518 | 1.929 |
| | **SiF - Uncertainty about the future** | 0.144 | 0.079 | 0.078 | 1.814 | 0.070 | 0.450 | 2.224 |
| | **Ruminations-brooding** | -0.211 | 0.128 | -0.085 | -1.655 | 0.099 | 0.321 | 3.113 |
| | **Ruminations-reflections** | -0.215 | 0.102 | -0.095 | -2.107 | 0.036 | 0.409 | 2.442 |
| | **Grit – consistency of interest** | 0.134 | 0.100 | 0.056 | 1.343 | 0.180 | 0.481 | 2.078 |
| | **Grit - perseverance of effort** | 0.384 | 0.110 | 0.142 | 3.490 | 0.001 | 0.505 | 1.981 |
| | **SSYSS - peers** | 0.090 | 0.089 | 0.041 | 1.008 | 0.314 | 0.517 | 1.935 |
| | **SSYSS - teacher** | -0.082 | 0.046 | -0.082 | -1.791 | 0.074 | 0.398 | 2.512 |
| | **SSYSS - parent** | 0.118 | 0.065 | 0.082 | 1.811 | 0.071 | 0.408 | 2.453 |
| | **PCQ - expectations** | 0.070 | 0.061 | 0.046 | 1.151 | 0.250 | 0.519 | 1.926 |
| | **PCQ - criticism** | -0.085 | 0.099 | -0.046 | -0.860 | 0.390 | 0.299 | 3.349 |
| | **PCQ - control** | -0.025 | 0.113 | -0.013 | -0.218 | 0.827 | 0.220 | 4.552 |
| | **PCQ – conditional regard** | 0.018 | 0.110 | 0.009 | 0.160 | 0.873 | 0.247 | 4.056 |
| | **PCQ - anxiousness** | -0.025 | 0.107 | -0.014 | -0.235 | 0.814 | 0.240 | 4.159 |
| | **RE – personal support from teachers** | 0.066 | 0.101 | 0.035 | 0.654 | 0.513 | 0.294 | 3.404 |
| | **RE – skill-focused support from teachers** | 0.032 | 0.173 | 0.009 | 0.186 | 0.853 | 0.353 | 2.830 |
| | **RE – personal support from peers** | 0.294 | 0.193 | 0.073 | 1.523 | 0.128 | 0.367 | 2.728 |
| | **RE – skill-focused support from peers** | -0.086 | 0.150 | -0.032 | -0.576 | 0.565 | 0.281 | 3.561 |
| | **participation in a school/university sports programme** | -0.039 | 0.138 | -0.016 | -0.285 | 0.776 | 0.281 | 3.557 |
| | **use of school/university** | 0.350 | 0.223 | 0.053 | 1.572 | 0.117 | 0.729 | 1.371 |

| | | | | | | | | |
|---|---|---|---|---|---|---|---|---|
| | **sports infrastructure** | | | | | | | |
| | **PVQ self-direction** | -0.014 | 0.161 | -0.003 | -0.085 | 0.932 | 0.693 | 1.443 |
| | **PVQ achievement** | 0.024 | 0.391 | 0.003 | 0.062 | 0.951 | 0.323 | 3.099 |
| | **PVQ hedonism** | 0.273 | 0.348 | 0.036 | 0.783 | 0.434 | 0.406 | 2.462 |
| | **PVQ recognition** | -0.125 | 0.388 | -0.016 | -0.322 | 0.747 | 0.326 | 3.066 |
| | **PVQ power** | 0.002 | 0.359 | 0.000 | 0.006 | 0.996 | 0.366 | 2.731 |
| | **PVQ security** | -0.080 | 0.348 | -0.010 | -0.229 | 0.819 | 0.410 | 2.442 |
| | **PVQ conformity** | -0.190 | 0.344 | -0.025 | -0.551 | 0.582 | 0.400 | 2.500 |
| | **PVQ tradition** | 0.341 | 0.325 | 0.046 | 1.052 | 0.293 | 0.441 | 2.267 |
| | **PVQ benevolence** | 0.485 | 0.353 | 0.061 | 1.376 | 0.170 | 0.429 | 2.333 |
| | **PVQ-universalism** | 0.078 | 0.420 | 0.010 | 0.186 | 0.853 | 0.283 | 3.535 |
| | **Gender** | -0.450 | 0.399 | -0.056 | -1.128 | 0.260 | 0.338 | 2.955 |

Table A2. Results of linear regression analysis on the recreational sample

| Model | | Unstandardized Coefficients | | Standardized Coefficients | | | Collinearity Statistics | |
|---|---|---|---|---|---|---|---|---|
| | | B | Std. Error | Beta | t | Sig. | Tolerance | VIF |
| 1 | (Constant) | 54.580 | 5.890 | | 9.266 | 0.000 | | |
| | Gender | 2.017 | 1.102 | 0.077 | 1.831 | 0.068 | 0.963 | 1.038 |
| | Age | -0.746 | 0.265 | -0.178 | -2.820 | 0.005 | 0.425 | 2.353 |
| | Level of study | 2.095 | 1.894 | 0.068 | 1.106 | 0.269 | 0.447 | 2.237 |
| | Type of settlement | 2.548 | 1.112 | 0.100 | 2.290 | 0.022 | 0.884 | 1.131 |
| | Mother's education | -1.443 | 1.131 | -0.062 | -1.276 | 0.203 | 0.716 | 1.397 |
| | Father's education | 0.650 | 1.119 | 0.028 | 0.581 | 0.562 | 0.711 | 1.407 |
| | Mother's employment | 0.347 | 1.763 | 0.009 | 0.197 | 0.844 | 0.879 | 1.137 |
| | Father's employment | 1.361 | 1.794 | 0.034 | 0.759 | 0.448 | 0.826 | 1.211 |
| | Change in family structure | -0.672 | 1.107 | -0.026 | -0.607 | 0.544 | 0.940 | 1.063 |
| | Having a sibling | 4.237 | 1.494 | 0.117 | 2.836 | 0.005 | 0.994 | 1.006 |
| 2 | (Constant) | 40.705 | 5.682 | | 7.164 | 0.000 | | |
| | Gender | -0.236 | 1.042 | -0.009 | -0.227 | 0.821 | 0.907 | 1.102 |
| | Age | -0.561 | 0.245 | -0.134 | -2.292 | 0.022 | 0.419 | 2.384 |
| | Level of study | 2.868 | 1.758 | 0.093 | 1.631 | 0.103 | 0.437 | 2.289 |
| | Type of settlement | 1.187 | 1.041 | 0.047 | 1.140 | 0.255 | 0.850 | 1.176 |
| | Mother's education | -1.561 | 1.048 | -0.067 | -1.490 | 0.137 | 0.703 | 1.423 |
| | Father's education | 0.679 | 1.029 | 0.030 | 0.660 | 0.510 | 0.709 | 1.411 |
| | Mother's employment | -0.114 | 1.620 | -0.003 | -0.070 | 0.944 | 0.877 | 1.141 |
| | Father's employment | -0.194 | 1.655 | -0.005 | -0.117 | 0.907 | 0.817 | 1.224 |
| | Change in family structure | -1.047 | 1.020 | -0.040 | -1.026 | 0.305 | 0.933 | 1.072 |
| | Having a sibling | 4.646 | 1.380 | 0.128 | 3.366 | 0.001 | 0.981 | 1.020 |
| | Exercise frequency | 2.232 | 0.553 | 0.169 | 4.034 | 0.000 | 0.813 | 1.231 |
| | Type of sport | 3.148 | 1.432 | 0.090 | 2.198 | 0.028 | 0.857 | 1.167 |
| | Sports club membership | 4.370 | 1.706 | 0.111 | 2.561 | 0.011 | 0.758 | 1.319 |
| | Training in a sports club (hours) | -0.191 | 0.206 | -0.039 | -0.926 | 0.355 | 0.799 | 1.252 |
| | Individual training (hours) | 1.104 | 0.158 | 0.317 | 6.976 | 0.000 | 0.694 | 1.442 |
| 3 | (Constant) | -4.532 | 6.094 | | -0.744 | 0.457 | | |
| | Gender | 0.431 | 0.860 | 0.016 | 0.501 | 0.617 | 0.757 | 1.321 |
| | Age | -0.320 | 0.189 | -0.076 | -1.695 | 0.091 | 0.401 | 2.491 |

| | | | | | | | | |
|---|---|---|---|---|---|---|---|---|
| | **Level of study** | -0.199 | 1.350 | -0.006 | -0.148 | 0.883 | 0.421 | 2.373 |
| | **Type of settlement** | 0.935 | 0.802 | 0.037 | 1.166 | 0.244 | 0.814 | 1.229 |
| | **Mother's education** | -1.293 | 0.813 | -0.056 | -1.591 | 0.112 | 0.665 | 1.504 |
| | **Father's education** | 0.925 | 0.791 | 0.040 | 1.170 | 0.243 | 0.682 | 1.467 |
| | **Mother's employment** | -0.765 | 1.234 | -0.019 | -0.620 | 0.536 | 0.860 | 1.163 |
| | **Father's employment** | 0.170 | 1.269 | 0.004 | 0.134 | 0.893 | 0.791 | 1.265 |
| | **Change in family structure** | -0.719 | 0.787 | -0.028 | -0.913 | 0.362 | 0.892 | 1.121 |
| | **Having a sibling** | 2.806 | 1.072 | 0.078 | 2.618 | 0.009 | 0.925 | 1.081 |
| | **Exercise frequency** | 1.111 | 0.428 | 0.084 | 2.596 | 0.010 | 0.772 | 1.295 |
| | **Type of sport** | 1.201 | 1.117 | 0.034 | 1.075 | 0.283 | 0.801 | 1.248 |
| | **Sports club membership** | 2.241 | 1.319 | 0.057 | 1.699 | 0.090 | 0.722 | 1.384 |
| | **Training in a sports club (hours)** | 0.009 | 0.161 | 0.002 | 0.055 | 0.956 | 0.749 | 1.334 |
| | **Individual training (hours)** | 0.621 | 0.125 | 0.178 | 4.974 | 0.000 | 0.634 | 1.577 |
| | **PSQ task-orientation** | 0.253 | 0.102 | 0.092 | 2.494 | 0.013 | 0.592 | 1.689 |
| | **PSQ ego-orientation** | 0.101 | 0.082 | 0.055 | 1.232 | 0.219 | 0.404 | 2.476 |
| | **SOQ win-orientation** | 0.010 | 0.111 | 0.005 | 0.093 | 0.926 | 0.277 | 3.606 |
| | **SOQ goal-orientation** | 1.252 | 0.170 | 0.373 | 7.359 | 0.000 | 0.317 | 3.155 |
| | **SOQ competition** | -0.011 | 0.183 | -0.003 | -0.060 | 0.952 | 0.346 | 2.887 |
| | **SAS-2 worry** | -0.036 | 0.138 | -0.013 | -0.263 | 0.793 | 0.311 | 3.211 |
| | **SAS-2-somatic anxiety** | -0.067 | 0.166 | -0.023 | -0.403 | 0.687 | 0.252 | 3.971 |
| | **SAS-2 concentration disruption** | 0.251 | 0.153 | 0.079 | 1.641 | 0.101 | 0.349 | 2.867 |
| | **Well-being** | 0.089 | 0.071 | 0.053 | 1.249 | 0.212 | 0.452 | 2.210 |
| | **SiF-positive future** | 0.259 | 0.098 | 0.092 | 2.649 | 0.008 | 0.679 | 1.472 |
| | **SiF – control of the future** | -0.059 | 0.125 | -0.022 | -0.475 | 0.635 | 0.372 | 2.687 |
| | **SiF - time management** | -0.013 | 0.138 | -0.004 | -0.098 | 0.922 | 0.465 | 2.152 |
| | **SiF-lack of self-efficacy** | 0.070 | 0.091 | 0.027 | 0.773 | 0.440 | 0.654 | 1.528 |
| | **SiF - Uncertainty about the future** | 0.015 | 0.103 | 0.006 | 0.150 | 0.881 | 0.436 | 2.293 |
| | **Ruminations-brooding** | -0.031 | 0.160 | -0.010 | -0.195 | 0.846 | 0.301 | 3.326 |

| | | | | | | | | |
|---|---|---|---|---|---|---|---|---|
| | **Ruminations-reflections** | 0.016 | 0.126 | 0.005 | 0.130 | 0.897 | 0.530 | 1.887 |
| | **Grit – consistency of interest** | 0.212 | 0.125 | 0.060 | 1.692 | 0.091 | 0.639 | 1.565 |
| | **Grit - perseverance of effort** | 0.699 | 0.137 | 0.194 | 5.089 | 0.000 | 0.557 | 1.795 |
| | **Gender** | 0.251 | 0.115 | 0.077 | 2.173 | 0.030 | 0.648 | 1.544 |
| **5** | **(Constant)** | -5.405 | 6.248 | | -0.865 | 0.387 | | |
| | **Gender** | 0.659 | 0.879 | 0.025 | 0.750 | 0.454 | 0.729 | 1.372 |
| | **Age** | -0.285 | 0.192 | -0.068 | -1.486 | 0.138 | 0.389 | 2.571 |
| | **Level of study** | -0.548 | 1.383 | -0.018 | -0.396 | 0.692 | 0.404 | 2.477 |
| | **Type of settlement** | 0.915 | 0.813 | 0.036 | 1.125 | 0.261 | 0.797 | 1.255 |
| | **Mother's education** | -1.504 | 0.826 | -0.065 | -1.822 | 0.069 | 0.648 | 1.544 |
| | **Father's education** | 0.915 | 0.799 | 0.040 | 1.146 | 0.252 | 0.673 | 1.486 |
| | **Mother's employment** | -0.624 | 1.249 | -0.016 | -0.500 | 0.617 | 0.844 | 1.185 |
| | **Father's employment** | 0.109 | 1.293 | 0.003 | 0.085 | 0.933 | 0.767 | 1.305 |
| | **Change in family structure** | -0.573 | 0.800 | -0.022 | -0.717 | 0.474 | 0.869 | 1.151 |
| | **Having a sibling** | 2.981 | 1.090 | 0.082 | 2.734 | 0.006 | 0.900 | 1.112 |
| | **Exercise frequency** | 1.068 | 0.433 | 0.081 | 2.465 | 0.014 | 0.758 | 1.319 |
| | **Type of sport** | 1.181 | 1.138 | 0.034 | 1.037 | 0.300 | 0.776 | 1.288 |
| | **Sports club membership** | 2.164 | 1.335 | 0.055 | 1.621 | 0.106 | 0.709 | 1.410 |
| | **Training in a sports club (hours)** | -0.025 | 0.163 | -0.005 | -0.151 | 0.880 | 0.730 | 1.369 |
| | **Individual training (hours)** | 0.650 | 0.129 | 0.186 | 5.056 | 0.000 | 0.601 | 1.664 |
| | **PSQ task-orientation** | 0.284 | 0.105 | 0.104 | 2.700 | 0.007 | 0.555 | 1.802 |
| | **PSQ ego-orientation** | 0.094 | 0.084 | 0.051 | 1.124 | 0.262 | 0.390 | 2.565 |
| | **SOQ win-orientation** | 0.001 | 0.112 | 0.000 | 0.005 | 0.996 | 0.273 | 3.660 |
| | **SOQ goal-orientation** | 1.225 | 0.173 | 0.364 | 7.099 | 0.000 | 0.310 | 3.224 |
| | **SOQ competition** | -0.003 | 0.187 | -0.001 | -0.016 | 0.987 | 0.331 | 3.022 |
| | **SAS-2 worry** | -0.030 | 0.143 | -0.011 | -0.211 | 0.833 | 0.294 | 3.402 |
| | **SAS-2-somatic anxiety** | -0.079 | 0.169 | -0.027 | -0.471 | 0.638 | 0.245 | 4.087 |
| | **SAS-2 concentration disruption** | 0.270 | 0.157 | 0.085 | 1.716 | 0.087 | 0.331 | 3.022 |
| | **Well-being** | 0.085 | 0.073 | 0.050 | 1.160 | 0.247 | 0.436 | 2.296 |

| | | | | | | | | |
|---|---|---|---|---|---|---|---|---|
| | **SiF-positive future** | 0.232 | 0.101 | 0.082 | 2.310 | 0.021 | 0.645 | 1.549 |
| | **SiF – control of the future** | -0.074 | 0.128 | -0.028 | -0.576 | 0.565 | 0.357 | 2.804 |
| | **SiF - time management** | -0.022 | 0.140 | -0.007 | -0.153 | 0.878 | 0.451 | 2.218 |
| | **SiF-lack of self-efficacy** | 0.072 | 0.092 | 0.028 | 0.777 | 0.438 | 0.638 | 1.568 |
| | **SiF - Uncertainty about the future** | -0.011 | 0.105 | -0.005 | -0.108 | 0.914 | 0.422 | 2.369 |
| | **Ruminations-brooding** | -0.019 | 0.162 | -0.006 | -0.116 | 0.907 | 0.295 | 3.392 |
| | **Ruminations-reflections** | 0.008 | 0.129 | 0.002 | 0.060 | 0.953 | 0.509 | 1.965 |
| | **Grit – consistency of interest** | 0.214 | 0.128 | 0.061 | 1.667 | 0.096 | 0.612 | 1.633 |
| | **Grit - perseverance of effort** | 0.668 | 0.139 | 0.186 | 4.791 | 0.000 | 0.544 | 1.837 |
| | **SSYSS - peers** | 0.234 | 0.118 | 0.072 | 1.984 | 0.048 | 0.624 | 1.603 |
| | **SSYSS - teacher** | 0.002 | 0.057 | 0.002 | 0.041 | 0.967 | 0.455 | 2.196 |
| | **SSYSS - parent** | 0.045 | 0.079 | 0.021 | 0.567 | 0.571 | 0.576 | 1.736 |
| | **PCQ - expectations** | 0.013 | 0.082 | 0.006 | 0.153 | 0.879 | 0.561 | 1.782 |
| | **PCQ - criticism** | 0.199 | 0.158 | 0.083 | 1.256 | 0.210 | 0.186 | 5.374 |
| | **PCQ - control** | -0.121 | 0.202 | -0.045 | -0.597 | 0.551 | 0.142 | 7.039 |
| | **PCQ – conditional regard** | 0.120 | 0.175 | 0.042 | 0.688 | 0.492 | 0.223 | 4.485 |
| | **PCQ - anxiousness** | -0.076 | 0.172 | -0.030 | -0.440 | 0.660 | 0.176 | 5.688 |
| | **RE – personal support from teachers** | -0.095 | 0.186 | -0.039 | -0.511 | 0.609 | 0.144 | 6.947 |
| | **RE – skill-focused support from teachers** | -0.054 | 0.219 | -0.011 | -0.246 | 0.806 | 0.424 | 2.360 |
| | **RE – personal support from peers** | -0.321 | 0.265 | -0.053 | -1.213 | 0.226 | 0.421 | 2.377 |
| | **RE – skill-focused support from peers** | 0.165 | 0.206 | 0.037 | 0.800 | 0.424 | 0.377 | 2.649 |
| | **participation in a school/university sports programme** | 0.177 | 0.185 | 0.046 | 0.957 | 0.339 | 0.355 | 2.815 |
| | **use of school/university sports infrastructure** | -5.597 | 6.292 | | -0.890 | 0.374 | | |
| | **Gender** | 0.609 | 0.882 | 0.023 | 0.691 | 0.490 | 0.726 | 1.378 |
| | **Age** | -0.259 | 0.194 | -0.062 | -1.334 | 0.183 | 0.380 | 2.631 |

| | | | | | | | | |
|---|---|---|---|---|---|---|---|---|
| | **Level of study** | -0.648 | 1.388 | -0.021 | -0.467 | 0.641 | 0.402 | 2.489 |
| | **Type of settlement** | 0.871 | 0.815 | 0.034 | 1.069 | 0.286 | 0.795 | 1.258 |
| | **Mother's education** | -1.497 | 0.830 | -0.064 | -1.803 | 0.072 | 0.642 | 1.559 |
| | **Father's education** | 0.936 | 0.800 | 0.041 | 1.170 | 0.243 | 0.672 | 1.488 |
| | **Mother's employment** | -0.579 | 1.251 | -0.014 | -0.462 | 0.644 | 0.842 | 1.187 |
| | **Father's employment** | 0.257 | 1.301 | 0.006 | 0.198 | 0.843 | 0.758 | 1.320 |
| | **Change in family structure** | -0.584 | 0.801 | -0.022 | -0.729 | 0.466 | 0.867 | 1.154 |
| | **Having a sibling** | 3.022 | 1.097 | 0.084 | 2.756 | 0.006 | 0.891 | 1.123 |
| | **Exercise frequency** | 1.070 | 0.434 | 0.081 | 2.465 | 0.014 | 0.757 | 1.321 |
| | **Type of sport** | 1.289 | 1.151 | 0.037 | 1.120 | 0.263 | 0.761 | 1.314 |
| | **Sports club membership** | 2.171 | 1.340 | 0.055 | 1.620 | 0.106 | 0.705 | 1.418 |
| | **Training in a sports club (hours)** | -0.023 | 0.163 | -0.005 | -0.143 | 0.887 | 0.730 | 1.370 |
| | **Individual training (hours)** | 0.670 | 0.131 | 0.192 | 5.107 | 0.000 | 0.578 | 1.731 |
| | **PSQ task-orientation** | 0.269 | 0.106 | 0.098 | 2.532 | 0.012 | 0.545 | 1.835 |
| | **PSQ ego-orientation** | 0.082 | 0.084 | 0.045 | 0.977 | 0.329 | 0.383 | 2.609 |
| | **SOQ win-orientation** | 0.007 | 0.113 | 0.003 | 0.062 | 0.951 | 0.272 | 3.675 |
| | **SOQ goal-orientation** | 1.219 | 0.173 | 0.363 | 7.052 | 0.000 | 0.310 | 3.230 |
| | **SOQ competition** | 0.019 | 0.189 | 0.005 | 0.102 | 0.919 | 0.327 | 3.060 |
| | **SAS-2 worry** | -0.034 | 0.143 | -0.013 | -0.237 | 0.813 | 0.293 | 3.408 |
| | **SAS-2-somatic anxiety** | -0.089 | 0.169 | -0.031 | -0.528 | 0.598 | 0.244 | 4.101 |
| | **SAS-2 concentration disruption** | 0.276 | 0.158 | 0.087 | 1.748 | 0.081 | 0.331 | 3.025 |
| | **Well-being** | 0.083 | 0.073 | 0.049 | 1.134 | 0.257 | 0.435 | 2.298 |
| | **SiF-positive future** | 0.235 | 0.101 | 0.083 | 2.328 | 0.020 | 0.645 | 1.551 |
| | **SiF – control of the future** | -0.070 | 0.128 | -0.026 | -0.545 | 0.586 | 0.354 | 2.822 |
| | **SiF - time management** | -0.024 | 0.140 | -0.007 | -0.172 | 0.863 | 0.451 | 2.219 |
| | **SiF-lack of self-efficacy** | 0.074 | 0.092 | 0.029 | 0.801 | 0.424 | 0.637 | 1.569 |
| | **SiF - Uncertainty about the future** | -0.015 | 0.105 | -0.006 | -0.141 | 0.888 | 0.421 | 2.373 |
| | **Ruminations-brooding** | -0.021 | 0.163 | -0.007 | -0.128 | 0.899 | 0.293 | 3.418 |

| | | | | | | | | |
|---|---|---|---|---|---|---|---|---|
| | **Ruminations-reflections** | 0.021 | 0.129 | 0.007 | 0.166 | 0.868 | 0.504 | 1.986 |
| | **Grit – consistency of interest** | 0.210 | 0.128 | 0.060 | 1.633 | 0.103 | 0.611 | 1.635 |
| | **Grit - perseverance of effort** | 0.662 | 0.140 | 0.184 | 4.740 | 0.000 | 0.543 | 1.843 |
| | **SSYSS - peers** | 0.230 | 0.118 | 0.071 | 1.951 | 0.052 | 0.623 | 1.605 |
| | **SSYSS - teacher** | 0.005 | 0.057 | 0.004 | 0.083 | 0.934 | 0.453 | 2.209 |
| | **SSYSS - parent** | 0.050 | 0.080 | 0.024 | 0.622 | 0.534 | 0.571 | 1.752 |
| | **PCQ - expectations** | 0.015 | 0.083 | 0.007 | 0.184 | 0.854 | 0.560 | 1.784 |
| | **PCQ - criticism** | 0.199 | 0.159 | 0.083 | 1.255 | 0.210 | 0.185 | 5.391 |
| | **PCQ - control** | -0.134 | 0.203 | -0.050 | -0.660 | 0.510 | 0.141 | 7.103 |
| | **PCQ – conditional regard** | 0.133 | 0.175 | 0.046 | 0.756 | 0.450 | 0.222 | 4.509 |
| | **PCQ - anxiousness** | -0.071 | 0.173 | -0.028 | -0.409 | 0.683 | 0.176 | 5.697 |
| | **RE – personal support from teachers** | -0.089 | 0.186 | -0.036 | -0.479 | 0.632 | 0.144 | 6.953 |
| | **RE – skill-focused support from teachers** | -0.031 | 0.221 | -0.006 | -0.142 | 0.887 | 0.419 | 2.385 |
| | **RE – personal support from peers** | -0.334 | 0.265 | -0.056 | -1.258 | 0.209 | 0.420 | 2.382 |
| | **RE – skill-focused support from peers** | 0.162 | 0.207 | 0.037 | 0.783 | 0.434 | 0.376 | 2.661 |
| | **participation in a school/university sports programme** | 0.181 | 0.185 | 0.047 | 0.977 | 0.329 | 0.355 | 2.817 |
| | **use of school/university sports infrastructure** | -0.395 | 0.382 | -0.037 | -1.033 | 0.302 | 0.623 | 1.604 |
| | **Gender** | 0.138 | 0.222 | 0.021 | 0.620 | 0.535 | 0.714 | 1.401 |
| **6** | **(Constant)** | -6.522 | 6.353 | | -1.027 | 0.305 | | |
| | **Gender** | 0.864 | 0.890 | 0.033 | 0.971 | 0.332 | 0.705 | 1.419 |
| | **Age** | -0.243 | 0.196 | -0.058 | -1.240 | 0.216 | 0.371 | 2.693 |
| | **Level of study** | -0.780 | 1.387 | -0.025 | -0.563 | 0.574 | 0.398 | 2.514 |
| | **Type of settlement** | 0.759 | 0.818 | 0.030 | 0.928 | 0.354 | 0.781 | 1.280 |
| | **Mother's education** | -1.539 | 0.841 | -0.066 | -1.829 | 0.068 | 0.618 | 1.618 |
| | **Father's education** | 1.153 | 0.806 | 0.050 | 1.430 | 0.153 | 0.655 | 1.527 |
| | **Mother's employment** | -0.591 | 1.261 | -0.015 | -0.469 | 0.639 | 0.821 | 1.218 |
| | **Father's employment** | 0.009 | 1.302 | 0.000 | 0.007 | 0.994 | 0.748 | 1.336 |

| | | | | | | | | |
|---|---|---|---|---|---|---|---|---|
| | **Change in family structure** | -0.692 | 0.808 | -0.027 | -0.856 | 0.393 | 0.843 | 1.186 |
| | **Having a sibling** | 2.761 | 1.103 | 0.076 | 2.503 | 0.013 | 0.871 | 1.149 |
| | **Exercise frequency** | 1.058 | 0.435 | 0.080 | 2.432 | 0.015 | 0.744 | 1.344 |
| | **Type of sport** | 1.130 | 1.150 | 0.032 | 0.983 | 0.326 | 0.754 | 1.326 |
| | **Sports club membership** | 2.268 | 1.344 | 0.058 | 1.688 | 0.092 | 0.693 | 1.442 |
| | **Training in a sports club (hours)** | -0.015 | 0.164 | -0.003 | -0.093 | 0.926 | 0.717 | 1.396 |
| | **Individual training (hours)** | 0.680 | 0.132 | 0.195 | 5.170 | 0.000 | 0.569 | 1.757 |
| | **PSQ task-orientation** | 0.262 | 0.106 | 0.096 | 2.473 | 0.014 | 0.541 | 1.849 |
| | **PSQ ego-orientation** | 0.086 | 0.086 | 0.047 | 0.996 | 0.320 | 0.363 | 2.751 |
| | **SOQ win-orientation** | 0.053 | 0.115 | 0.026 | 0.459 | 0.646 | 0.256 | 3.909 |
| | **SOQ goal-orientation** | 1.176 | 0.175 | 0.350 | 6.706 | 0.000 | 0.297 | 3.365 |
| | **SOQ competition** | -0.002 | 0.189 | -0.001 | -0.011 | 0.991 | 0.322 | 3.110 |
| | **SAS-2 worry** | -0.035 | 0.145 | -0.013 | -0.241 | 0.810 | 0.285 | 3.509 |
| | **SAS-2-somatic anxiety** | -0.110 | 0.170 | -0.038 | -0.647 | 0.518 | 0.239 | 4.176 |
| | **SAS-2 concentration disruption** | 0.307 | 0.158 | 0.097 | 1.945 | 0.052 | 0.327 | 3.061 |
| | **Well-being** | 0.112 | 0.074 | 0.066 | 1.509 | 0.132 | 0.421 | 2.377 |
| | **SiF-positive future** | 0.181 | 0.103 | 0.064 | 1.752 | 0.080 | 0.610 | 1.640 |
| | **SiF – control of the future** | -0.083 | 0.131 | -0.031 | -0.635 | 0.526 | 0.336 | 2.978 |
| | **SiF - time management** | -0.103 | 0.144 | -0.031 | -0.719 | 0.472 | 0.426 | 2.350 |
| | **SiF-lack of self-efficacy** | 0.085 | 0.095 | 0.033 | 0.898 | 0.369 | 0.598 | 1.673 |
| | **SiF - Uncertainty about the future** | -0.048 | 0.105 | -0.020 | -0.452 | 0.651 | 0.412 | 2.425 |
| | **Ruminations-brooding** | -0.046 | 0.166 | -0.015 | -0.279 | 0.780 | 0.278 | 3.600 |
| | **Ruminations-reflections** | 0.042 | 0.130 | 0.013 | 0.321 | 0.749 | 0.491 | 2.039 |
| | **Grit – consistency of interest** | 0.203 | 0.130 | 0.058 | 1.560 | 0.119 | 0.591 | 1.693 |
| | **Grit - perseverance of effort** | 0.628 | 0.141 | 0.175 | 4.464 | 0.000 | 0.528 | 1.893 |
| | **SSYSS - peers** | 0.265 | 0.119 | 0.081 | 2.224 | 0.027 | 0.604 | 1.657 |
| | **SSYSS - teacher** | 0.009 | 0.057 | 0.007 | 0.164 | 0.870 | 0.443 | 2.257 |
| | **SSYSS - parent** | 0.036 | 0.080 | 0.017 | 0.446 | 0.656 | 0.560 | 1.784 |

| | | | | | | | | |
|---|---|---|---|---|---|---|---|---|
| | **PCQ - expectations** | 0.012 | 0.083 | 0.006 | 0.150 | 0.881 | 0.552 | 1.810 |
| | **PCQ - criticism** | 0.178 | 0.159 | 0.075 | 1.118 | 0.264 | 0.182 | 5.499 |
| | **PCQ - control** | -0.142 | 0.203 | -0.053 | -0.697 | 0.486 | 0.139 | 7.206 |
| | **PCQ – conditional regard** | 0.144 | 0.180 | 0.050 | 0.801 | 0.423 | 0.208 | 4.815 |
| | **PCQ - anxiousness** | -0.078 | 0.172 | -0.031 | -0.453 | 0.651 | 0.174 | 5.745 |
| | **RE – personal support from teachers** | -0.068 | 0.187 | -0.028 | -0.365 | 0.715 | 0.140 | 7.117 |
| | **RE – skill-focused support from teachers** | -0.070 | 0.223 | -0.014 | -0.312 | 0.755 | 0.404 | 2.474 |
| | **RE – personal support from peers** | -0.344 | 0.267 | -0.057 | -1.290 | 0.197 | 0.410 | 2.437 |
| | **RE – skill-focused support from peers** | 0.152 | 0.210 | 0.034 | 0.725 | 0.469 | 0.361 | 2.772 |
| | **participation in a school/university sports programme** | 0.159 | 0.187 | 0.041 | 0.852 | 0.395 | 0.344 | 2.905 |
| | **use of school/university sports infrastructure** | -0.380 | 0.383 | -0.036 | -0.991 | 0.322 | 0.613 | 1.632 |
| | **PVQ self-direction** | 0.159 | 0.222 | 0.024 | 0.715 | 0.475 | 0.707 | 1.415 |
| | **PVQ achievement** | 0.470 | 0.488 | 0.043 | 0.962 | 0.336 | 0.402 | 2.489 |
| | **PVQ hedonism** | 0.630 | 0.386 | 0.066 | 1.633 | 0.103 | 0.501 | 1.997 |
| | **PVQ recognition** | 0.311 | 0.473 | 0.029 | 0.659 | 0.510 | 0.420 | 2.384 |
| | **PVQ power** | -0.017 | 0.420 | -0.002 | -0.040 | 0.968 | 0.458 | 2.184 |
| | **PVQ security** | -0.867 | 0.388 | -0.087 | -2.234 | 0.026 | 0.533 | 1.877 |
| | **PVQ conformity** | 0.182 | 0.422 | 0.018 | 0.431 | 0.667 | 0.475 | 2.104 |
| | **PVQ tradition** | 0.429 | 0.397 | 0.042 | 1.081 | 0.280 | 0.545 | 1.836 |
| | **PVQ benevolence** | -0.078 | 0.397 | -0.008 | -0.197 | 0.844 | 0.515 | 1.941 |
| | **PVQ-universalism** | -0.988 | 0.540 | -0.092 | -1.830 | 0.068 | 0.321 | 3.114 |
| | **Gender** | 0.579 | 0.524 | 0.053 | 1.104 | 0.270 | 0.356 | 2.809 |
